# Fluorescent RET-Based Chemosensor Bearing 1,8-Naphthalimide and Styrylpyridine Chromophores for Ratiometric Detection of Hg^2+^ and Its Bio-Application

**DOI:** 10.3390/bios12090770

**Published:** 2022-09-19

**Authors:** Pavel A. Panchenko, Anastasija V. Efremenko, Anna S. Polyakova, Alexey V. Feofanov, Maria A. Ustimova, Yuri V. Fedorov, Olga A. Fedorova

**Affiliations:** 1 Laboratory of Photoactive Supramolecular Systems, A. N. Nesmeyanov Institute of Organoelement Compounds of Russian Academy of Sciences, Vavilova Str. 28, 119991 Moscow, Russia; 2 Department of Technology of Fine Organic Synthesis and Chemistry of Dyes, Dmitry Mendeleev University of Chemical Technology of Russia, Miusskaya Sqr. 9, 125047 Moscow, Russia; 3 Biological Faculty, Lomonosov Moscow State University, Leninskie Gory 1, 119992 Moscow, Russia; 4 Laboratory of Optical Microscopy and Spectroscopy, Shemyakin-Ovchinnikov Institute of Bioorganic Chemistry of Russian Academy of Sciences, Miklukho-Maklaya Str. 16/10, 117997 Moscow, Russia

**Keywords:** naphthalimide, styryl dye, crown ether, click-reaction, mercury (II) cation, A549 cells, HEK293 cells, ratiometric probe, sensor, fluorescence imaging, resonance energy transfer, photoinduced electron transfer

## Abstract

Dyad compound **NI-SP** bearing 1,8-naphthalimide (NI) and styrylpyridine (SP) photoactive units, in which the N-phenylazadithia-15-crown-5 ether receptor is linked with the energy donor naphthalimide chromophore, has been evaluated as a ratiometric fluorescent chemosensor for mercury (II) ions in living cells. In an aqueous solution, **NI-SP** selectively responds to the presence of Hg^2+^ via the enhancement in the emission intensity of NI due to the inhibition of the photoinduced electron transfer from the receptor to the NI fragment. At the same time, the long wavelength fluorescence band of SP, arising as a result of resonance energy transfer from the excited NI unit, appears to be virtually unchanged upon Hg^2+^ binding. This allows self-calibration of the optical response. The observed spectral behavior is consistent with the formation of the (**NI-SP**)·Hg^2+^ complex (dissociation constant 0.13 ± 0.04 µM). Bio-imaging studies showed that the ratio of fluorescence intensity in the 440–510 nm spectral region to that in the 590–650 nm region increases from 1.1 to 2.8 when cells are exposed to an increasing concentration of mercury (II) ions, thus enabling the detection of intracellular Hg^2+^ ions and their quantitative analysis in the 0.04–1.65 μM concentration range.

## 1. Introduction

Heavy and transition metals’ contamination of the environment causes serious health problems to humans. Mercury pollution specifically is an important global concern since it originates from a variety of natural and anthropogenic sources including oceanic and volcanic emission, gold mining, solid waste incineration and the combustion of fossil fuels [1,2,3]. Dangerous levels of Hg(II) can enter the organism through the consumption of large edible fish, where this toxin is accumulated [4]. In the human body, mercury can induce neurological diseases, kidney failure and gastrointestinal organ damage [5,6]. As such, the development of sensor devices capable of detecting Hg^2+^ cation in water and in biological samples is currently in great demand.

Fluorescence spectroscopy is a powerful technique for the quantification of low concentrations of analytes. To date, a large number of fluorescence-based small-molecule probes for Hg(II) have been reported. Most of them demonstrate OFF→ON or ON→OFF switching of the emission intensity by the PET (photoinduced electron transfer) mechanism [7,8,9,10] or as a result of Hg(II)-dependent irreversible chemical reactions [11,12,13]. Such measurements based on intensity changes are easily influenced by external factors, including concentration variations and the intensity of excitation. By contrast, measuring optical signals as intensity ratios at two different wavelengths provides a built-in correction for the environmental effects and may assuage many of the problems associated with intensity-based sensors [14]. Despite the obvious advantage, the number of ratiometric fluorescent probes for Hg^2+^ cation is rather limited even for in vitro analysis, and most of them exploit the Hg^2+^-induced spyrolactam ring opening of rhodamine dyes, which act as energy acceptor chromophores [15,16,17].

**Scheme 1:**
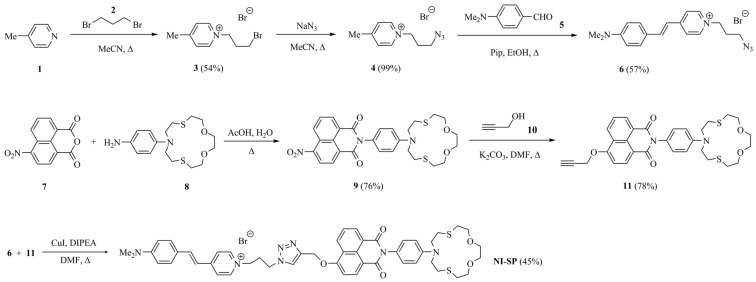
Synthesis of compound **NI-SP**.

Herein, we present the design, synthesis, spectral behavior and living cell application of a fluorescent chemosensor **NI**-**SP** (Scheme 1). In the **NI**-**SP** structure, naphthalimide (NI) and styrylpyridine (SP) chromophores are linked through a spacer group. The choice of SP fragment was due to its relatively long wavelength emission (*ca*. 600 nm) [18] as well as the presence of a positive charge in the pyridinium core, which is supposed to enhance water compatibility. Naphthalimides are classic fluorescent dyes whose electronic absorption and emission depend on the properties of the molecular structure and surrounding medium [19,20,21,22]; they have been widely applied as photoactive components in the construction of molecular devices with switchable optical characteristics [23,24,25,26] including fluorescent chemosensors [27,28,29,30]. As a receptor, we used azadithia-15-crown-5 (A15C5) ether exhibiting high affinity towards mercury (II) cations in a slightly acidic aqueous solution [18,31,32]. It has recently been shown that the presence of the A15C5 group in the *N*-aryl substituent at imide nitrogen of the 1,8-naphthalimide residue results in OFF→ON fluoroionophores based on the PET signaling mechanism [31,32]. The same structural feature was realized in the case of the **NI**-**SP** probe, thereby providing for the NI fragment to sense cations via the increase in its fluorescence intensity. Additionally, resonance energy transfer (RET) from the donor NI to the acceptor SP chromophore in **NI**-**SP** produced the second emission band essential for the self-calibration of the optical response.

## 2. Materials and Methods

### 2.1. Materials

Experimental details concerning the syntheses of compounds **4**, **6**, **11** and **NI**-**SP** (Scheme 1) are presented in the Supplementary Materials. Acetate buffer solution was prepared with deionized water (18.2 MΩ·cm). Perchlorates of Ag^+^, Zn^2+^, Cu^2+^, Pb^2+^, Ni^2+^, Cd^2+^, Fe^2+^, Mg^2+^ and Ca^2+^ were dissolved in MeCN (HPLC grade) and then used in spectroscopic studies. Hg(ClO_4_)_2_ was dissolved in water and stabilized by the addition of 0.5 equiv. HClO_4_. The exact concentration of Hg(ClO_4_)_2_ was determined by complexometric titration using EDTA and xylenol orange as an indicator.

### 2.2. Optical Spectroscopy

Absorption and fluorescence spectra of the compounds were recorded in air-saturated solutions at ambient temperature with a Cary 300 spectrophotometer (Agilent Technologies, Santa Clara, CA, USA) and FluoroLog-3-221 spectrofluorometer (Horiba Jobin Yvon, Edison, NJ, USA). All measured fluorescence spectra were corrected for the nonuniformity of detector spectral sensitivity. Coumarin 481 in acetonitrile (fluorescence quantum yield is 0.08) [33] was used as a reference for the fluorescence quantum yield measurements.

### 2.3. Equilibrium Constants’ Determination

**Scheme 2:**
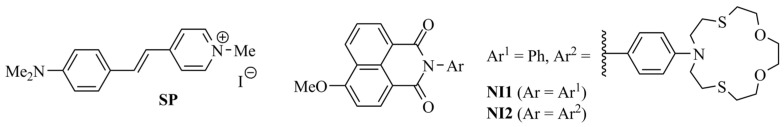
Chemical structure of compounds **NI1, NI2** and **SP**.

Complex formation of ligands **NI2** (Scheme 2) and **NI-SP** with Hg^2+^ was studied in aqueous solution by spectrofluorometric titration [34,35]. Compound **NI2** (or **NI**-**SP**) of known concentration in acetate buffer (pH 4.5, 0.1 M) was titrated with a solution of mercury (II) perchlorate. After addition of each aliquot of Hg(ClO_4_)_2_, fluorescence spectrum was recorded, and the stability constants of the complexes were determined using the SPECFIT/32 program (Spectrum Software Associates, West Marlborough, MA, USA). The following equilibria were considered in the fitting of experimental data

$$L+{\mathrm{Hg}}^{2+}⇄\left(L\right)\cdot {\mathrm{Hg}}^{2+}$$

$$2L+{\mathrm{Hg}}^{2+}⇄{\left(L\right)}_{2}\cdot {\mathrm{Hg}}^{2+}$$

In doing so, it was found that the experimental data corresponded to the theoretical data if only the first equilibrium was taken into account, and the formation of the complex with composition of 2:1 was assumed to be negligible.

### 2.4. Computational Details

The three dimensional structures of **NI-SP** and its frontier MOs were obtained with MOPAC 2016 program package using PM6 semiempirical method [36]. The calculations were performed at optimized geometries, which reached gradient variations less than 0.01 kcal/mol. The solvent effect was included in geometry optimizations following the conductor-like screening model (COSMO) implemented in MOPAC. A dielectric constant of *ε* = 80 and a refraction index of solvent (*n*) such that *n*^2^ = 2 were used.

### 2.5. Cells and Their Treatment

Human lung adenocarcinoma cells A549 and human embryonic kidney 293 cells (HEK 293) were obtained from Ivanovsky institute of Virology (Russia) and grown (37 °C, 5% CO_2_) in DMEM Eagle medium supplemented with L-glutamine (2 mM) and 10% fetal bovine serum (i.e., a complete medium). Cells were subcultured two times per week. For microscopic experiments, cells were seeded (2 × 10^5^ cells per ml, per well) on round cover glasses placed in 24-well plates and grown for 24 h.

To study cellular accumulation, distribution and uptake kinetics of **NI-SP**, cells were incubated in a complete medium (5–120 min, 37 °C) with **NI-SP** (5–10 µM) added from a 1 mM stock solution in dimethyl sulfoxide, washed twice with Hanks’ solution and subjected to microscopy analysis. Retention of **NI-SP** in cells was studied for cells pre-incubated with **NI-SP** (10 µM) for 60 min, placed in a fresh medium without **NI-SP** for different (0–4.5 h) periods of time and recorded with the confocal laser scanning microscope at the identical parameters of measurements. Concentration dependence of **NI-SP** uptake was measured after 0.5 h incubation of cells with the compound. Fluorescence intensity (*I*_fl_) of **NI-SP** in cells was recorded in the 420–730 nm spectral range, averaged over 40–50 cells and presented as the mean value ± SEM.

For the study of intracellular complexation of Hg^2+^, Cu^2+^ or Pb^2+^ with **NI-SP**, the cells were pre-incubated (20–40 min) with either Hg(ClO_4_)_2_ at 2–50 µM, or Pb(ClO_4_)_2_ (or Cu(ClO_4_)_2_) at 2 µM–10 mM. Next, the cells were washed twice with Hanks’ solution, incubated (20 min) with **NI-SP** at 10 µM, washed twice with Hanks’ solution and subjected to microscopy measurements. For the study of intracellular complexation of Ag^+^ with **NI-SP**, the cells were pre-incubated with Ag(ClO_4_) (2–500 µM, 10–60 min) or with Hg(ClO_4_)_2_ (10 µM) and Ag(ClO_4_) (2–500 µM) for 10–30 min. Next, the cells were washed twice with Hanks’ solution, incubated with **NI-SP** (10 µM, 20 min) washed twice with Hanks’ solution and subjected to microscopy measurements.

It should be mentioned that the used regimes of incubation of A549 cells with **NI-SP** (10 µM) and Hg(ClO_4_)_2_ (2–50 µM) did not induce death of cells as verified by the live/dead cell assay based on differential ability of Hoechst 33,342 and propidium iodide to penetrate in living and dead cells (data not present). Cytotoxicity of **NI-SP** and Hg(ClO_4_)_2_ for A549 cells was additionally estimated after 24 and 48 h incubation of cells with **NI-SP** or Hg(ClO_4_)_2_ separately using MTT assay.

### 2.6. Confocal Microscopy Measurements

Fluorescence microscopy studies of **NI-SP** were performed with the LSM-710 confocal laser scanning microscope (Carl Zeiss AG, Oberkochen, Germany). The confocal fluorescent images were obtained with the α-Plan-Apochromat 100×/1.4 oil-immersion objective at 0.3 µm lateral and 1.5 µm axial resolution. Studying the intracellular distribution of **NI-SP** or the kinetics of cellular accumulation/retention of **NI-SP** in cells, fluorescence was excited at the 405 nm wavelength, and emission was registered in the 440–710 nm or 440–510 and 590–650 nm spectral ranges. Alternatively, the 488 nm excitation wavelength and the 510–730 nm detection spectral range were used in the studies of intracellular distribution of **NI-SP**. For a ratiometric analysis (intracellular complexation of **NI-SP** with Hg^2+^, Cu^2+^, or Pb^2+^), fluorescence was excited at the 405 nm wavelength and recorded simultaneously in the 440–510 and 590–650 nm spectral ranges. Intracellular fluorescence spectra of **NI-SP** were recorded using the spectral mode of confocal image measurements: fluorescence was excited at the 405 or 488 nm wavelength and recorded in the 440–730 or 510–730 nm spectral ranges with a spectral resolution of 5 nm.

## 3. Results and Discussion

### 3.1. Synthesis of Chemosensor NI-SP

For the preparation of chemosensor **NI**-**SP**, we used Cu(I)-catalyzed azide-alkyne cycloaddition click-reaction between the styryl derivative (**6**) and crown-containing 4-propargyloxy-1,8-naphthalimide (**11**) (Scheme 1). The former was obtained by the condensation of 1-(3-azidopropyl)-4-methylpyridinium bromide (**4**) with *N*,*N*-dimethyl- aminobenzaldehyde (**5**) in basic conditions. The reaction of (**3**) with NaN_3_ to yield (**4**) proceeded with a moderate yield according to previously published protocol used for the conversion of 1-(3-iodopropyl)-4-methylpyridinium iodide to the corresponding azido derivative [37]. Alkylation of *γ*-picoline (**1**) with 1,3-dibromopropane (**2**) was performed in acetonitrile at reflux as described in reference [38]. To synthesize the propargyl derivative (**11**), we started from the imidation of 4-nitronaphthalic anhydride (**7**) with crown-containing arylamine (**8**) [39,40]. The isolated product (**9**) was further subjected to nucleophilic substitution of the nitro group at the C-4 position of the naphthalene ring under the treatment with propargyl alcohol and potassium carbonate in DMF.

### 3.2. Spectroscopic Characterization of the Free Ligand NI-SP

**Figure 1:**
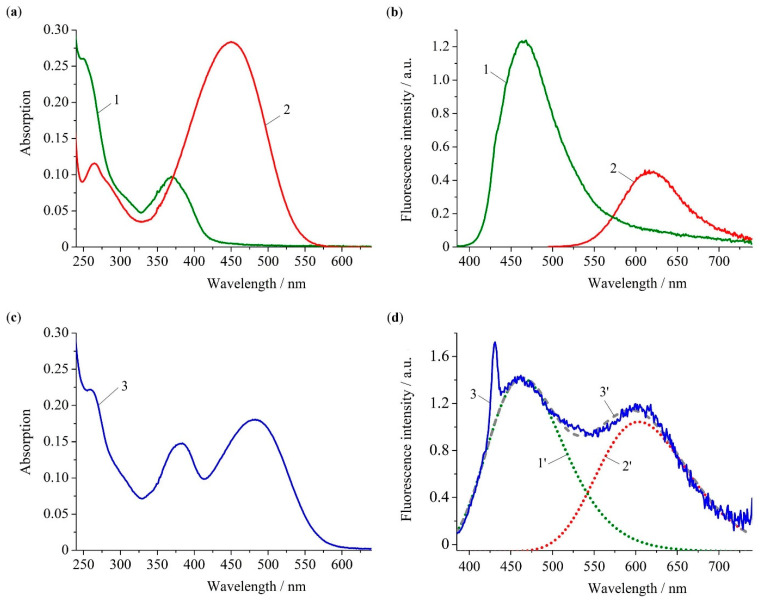
Absorption (**a**,**c**) and fluorescence (**b**,**d**) spectra of compounds **NI2** (1), **SP** (2*)* and **NI-SP** (3) in water. Excitation wavelengths are 375 nm (for **NI2** and **NI-SP**) and 455 nm (for **SP**). Concentrations of all compounds are 10.0 μM. Dashed line (3′) in panel (**d**) represents the fitting curve, which is a superposition of emission peaks of NI (1′) and SP (2′) fragments.

Steady-state absorption and emission characteristics of **NI-SP** were investigated in an aqueous solution. Previously obtained monochromophoric derivatives **NI1** [41], **NI2** [40] and **SP** [42] (Scheme 2) were used for the comparative analysis of spectral behavior. Optical properties of the studied compounds are presented in Table 1 and Figure 1.

**Figure 2:**
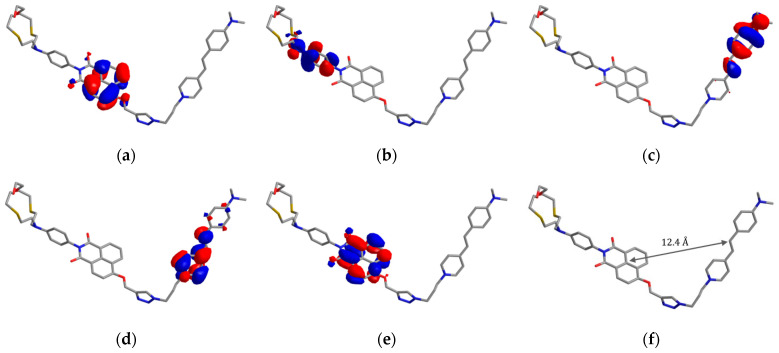
Frontier molecular orbitals (**a**–**e**) and optimized ground state geometry (**f**) of the compound **NI-SP** calculated by the PM6 method**.** (**a**) HOMO(–2), *E* = –9.13 eV; (**b**) HOMO(–1), *E* = –8.85 eV; (**c**) HOMO, *E* = –8.57 eV; (**d**) LUMO, *E* = –1.91 eV; (**e**) LUMO(+1), *E* = –1.49 eV.

Light absorption by the NI fragment in probe **NI**-**SP** can be attributed to the peak centered at 382 nm (Figure 1c), which is confirmed by the fact that model compounds **NI1** and **NI2** show the very close position of the long wavelength absorption maxima (Figure 1a, Table 1) resulting from the polarization of the naphthalimide chromophore. The fluorescence spectra of **NI1** and **NI2** are located in the visible region with maxima at 465 and 467 nm (Figure 1b, Table 1). A similar peak is observed in the **NI**-**SP** emission spectrum recorded upon excitation with light at 375 nm (Figure 1d). Compound **SP** demonstrates charge transfer (CT) absorption and emission bands with $\lambda_{\max}^{\mathrm{abs}}$ and $\lambda_{\max}^{\mathrm{fl}}$ at 450 nm and 619 nm, respectively. The **NI**-**SP** spectra also show peaks of the styrylpyridine unit; however, its $\lambda_{\max}^{\mathrm{abs}}$ was found to be red-shifted by 30 nm relative to that of **SP**. The latter observation could be a result of a rather high sensitivity of the positively charged SP chromophore to the microenvironment [43]. As can be seen from the optimized ground state geometry of **NI**-**SP** (see Figure 2f), the distance between the NI and SP groups could be as high as 12 Å. Such a distance is comparable with the dye molecules’ size. Hence, an overlap of electronic shells of the two photoactive fragments is possible to some extent. This in turn could change the energy of CT transition.

When excited at 375 nm, probe **NI**-**SP** clearly showed the presence of two bands in the fluorescence spectrum (Figure 1d). Considering the values of $\lambda_{\max}^{\mathrm{abs}}$ for compounds **NI2** and **SP**, the observed dual emission character of **NI**-**SP** spectrum can be explained by the direct excitation of both chromophores. On the other hand, the emission bands of dyes **NI1** and **NI2** overlap with the absorption of **SP** (see Supplementary Materials, Figure S1). Therefore, fluorescence at 605 nm may in part be a result of RET from NI to SP unit. To verify our assumption as to whether resonance energy transfer in the bichromophoric system **NI**-**SP** really occurs, we compared the fluorescence quantum yield of the energy donor naphthalimide fragment in **NI**-**SP** ($\varphi_{D}^{\mathrm{fl}}$) with that taken part of the energy acceptor ($\varphi_{D,0}^{\mathrm{fl}}$). As a $\varphi_{D,0}^{\mathrm{fl}}$ value, we chose the quantum yield of **NI2** (0.0073, see Table 1), whereas $\varphi_{D}^{\mathrm{fl}}$ was estimated using the absorption and fluorescence spectra of **NI**-**SP** (Figure 1c,d). In this estimation, it was allowed that (i) 50% of integrated dual emission intensity comes from naphthalimide (calculated as a ratio of areas underneath curves 1′ and 3′ in Figure 1d) and (ii) the NI fragment in **NI**-**SP** absorbs approximately two-thirds of the excitation light (roughly deduced from the comparison of absorption spectra depicted in Figure 1a,c). The calculation of $\varphi_{D}^{\mathrm{fl}}$ for the free ligand **NI**-**SP** thus gave the value equal to 0.00068. Using the obtained quantum yields $\varphi_{D}^{\mathrm{fl}}$ and $\varphi_{D,0}^{\mathrm{fl}}$, and Equation (1) [44],

(1) $$\Phi_{\mathrm{RET}}=1-\frac{\varphi_{D}^{\mathrm{fl}}}{\varphi_{D,0}^{\mathrm{fl}}}$$

the resonance energy transfer efficiency ($\Phi_{\mathrm{RET}}$) was found to be 0.91 (91%). The presented analysis shows that the main deactivation pathway for the excited NI chromophore in the ligand **NI-SP** is the resonance energy transfer. Alternatively, NI may relax through the PET from the *N*-aryl group bearing the crown ether substituent. The possibility of PET is confirmed by the quenching of the fluorescence of *N*-phenyl-1,8-naphthalimide **NI1** upon the introduction of the A15C5-group (compare $\varphi^{\mathrm{fl}}$ for **NI1** and **NI2** in Table 1) as well as by the calculations of frontier molecular orbitals of **NI-SP** using the PM6 semiempirical method (Figure 2a–e). One can see that the energy level of the highest occupied molecular orbital localized over the *N*-aryl fragment (−8.85 eV, HOMO(–1), Figure 2b) is higher than that of the local HOMO of naphthalimide (−9.13 eV, HOMO(–2), Figure 2a). Since the latter appears to be singly occupied once the donor chromophore has absorbed a photon and a HOMO(–2)→LUMO(+1) transition has occurred, thermodynamically feasible photoinduced electron transfer from the HOMO(–1) could be expected. It should be noted that the excited state of the acceptor SP fragment could hardly be quenched by the PET from the receptor group as its local HOMO lies lower than the frontier orbitals of styrylpyridine (−8.57 and −1.91 eV HOMO and LUMO, Figure 2c,d). Furthermore, the fluorescence quantum yield of the SP fragment in **NI-SP** (0.00049), estimated by analogy with the way used for the donor chromophore (see above), was rather close to the $\varphi^{\mathrm{fl}}$ value of the compound SP (0.00039, see Table 1).

### 3.3. Complex Formation Studies and Sensing Mechanism

As a medium for the study of **NI-SP** probe coordination with Hg^2+^ cations, we used 0.1 M aqueous acetate buffer solution (obtained by mixing AcOH and AcONa) with pH 4.5. The choice of such a pH level was due to our pursuit of following the complexation effects in conditions ultimately close to those realized in biological experiments (see Section 3.4). It has been subsequently found that **NI-SP** accumulates in lysosomes of human lung adenocarcinoma A549 cells (a cell line used for in vitro fluorescence imaging), where pH is rather acidic (4.5–4.8) [45]. We have also shown in our recent papers that AC15C5 ether fragment in the compound **NI2** [40], as well as in the naphthalimides with a similar structure but bearing NH_2_ [31] and NHAc [32] groups instead of OMe at the C-4 atom of the naphthalene core, starts to protonate at a pH below 4.0. Higher pH values thus provide a possibility for the switching of PET upon the interaction of the A15C5 macrocycle nitrogen lone pair with a metal cation. Indeed, we observed such switching in the case of compound **NI2** at chosen conditions (pH 4.5) through a significant Hg^2+^-induced fluorescence enhancement (Figure S2).

**Figure 3:**
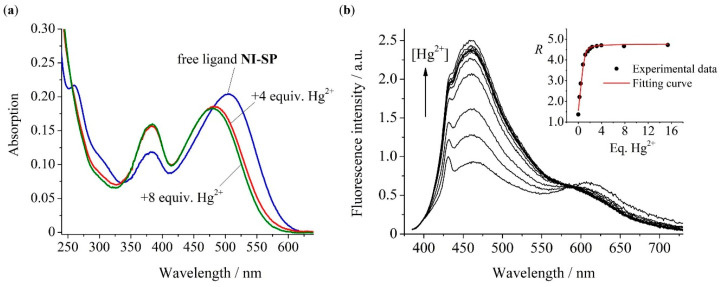
Changes in the absorption (**a**) and fluorescence (**b**) spectra of compound **NI**-**SP** (10.0 μM) upon addition of Hg(ClO_4_)_2_ at pH 4.5 (acetate buffer, 0.1 M). Excitation wavelength is 375 nm. The insert in panel (**b**) shows the ratio (*R*) of the emission intensity at 465 nm to that at 610 nm versus equivalents of mercury (II) perchlorate in the solution.

The addition of mercury (II) perchlorate to an aqueous solution of sensor **NI-SP** did not cause pronounced changes in the absorption spectrum (Figure 3a); however, it produced a 2.7-fold increase in NI chromophore emission intensity at 465 nm (Figure 3b), which is related to the inhibition of PET in the complex.

At the same time, the band of the SP fragment at 610 nm was virtually unchanged. This obviously affords dual emission ratiometric measurements by a comparison of the ratio of the fluorescence intensities at 465 nm and 610 nm (*I*_465_/*I*_610_) in the presence (*R*) and absence (*R*_F_) of Hg(II) cations. The observed changes in the emission spectrum of **NI-SP** were reversible (as shown by the alternant additions of Hg(ClO_4_)_2_ and EDTA, see Figure S3) and were consistent with the formation of a complex with a 1:1 metal to ligand ratio (see the insert in Figure 3b). Based on the spectrofluorimetric titration data, we calculated the logarithm of (**NI-SP**)·Hg^2+^ stability constant (lg *K*) to be 6.89 ± 0.13. The same complex composition and a rather close lg *K* value (7.62 ± 0.17) were found for the monochromophoric chemosensor **NI2** (Figure S2).

Analysis of the sensor responses of compounds **NI**-**SP** and **NI2** showed that the fluorescence enhancement at 465 nm (*FE*_465_), which is a ratio of the emission intensity after the addition of 5 equiv. Hg^2+^ to that of the free ligand, calculated from the **NI**-**SP** spectra (*FE*_465_ = 2.7, Figure 3b) was lower than in the case of **NI2** (*FE*_465_ = 28.5, Figure S2b). This difference can be rationalized if one takes into account that the radiative deactivation of the NI chromophore in the complex of **NI**-**SP** with Hg^2+^ cation is quenched by a resonance energy transfer. The RET efficiency in the complex (**NI**-**SP**)·Hg^2+^ was found in accordance with Equation (1). To calculate $\Phi_{\mathrm{RET}}$ we supposed that the $\varphi_{D}^{\mathrm{fl}}$ value of the NI unit in **NI**-**SP** is increased by a factor of 2.7 (*FE*_465_) as a result of complex formation. Apparently, *FE*_465_ reflects the ratio of fluorescence quantum yields before and after the addition of Hg^2+^ because NI absorption remains the same upon Hg^2+^ coordination (see Figure S2a, some hyperchromic effect at 380 nm in the **NI**-**SP** spectrum (Figure 3a) is probably due to a slight blue shift of the SP band). Assuming this finding along with an assumption that the quantum yield $\varphi_{D,0}^{\mathrm{fl}}$ (see Section 3.2) is as high as for the *N*-phenyl derivative **NI1** (0.71, Table 1), in which PET and RET processes do not occur, the $\Phi_{\mathrm{RET}}$ for (**NI**-**SP**)·Hg^2+^ appears to be 0.997 (99.7%). Theoretical calculations of $\Phi_{\mathrm{RET}}$ for a pair of dyes **NI1** and **SP** fixed at a distance 12.4 Å by the Förster model (see Supplementary Materials) testify to the same result (99.97%). Notably, the calculation of $\Phi_{\mathrm{RET}}$ for (**NI**-**SP**)·Hg^2+^ by Equation (1) can also be performed if the emission quantum yield of the complex (**NI2**)·Hg^2+^ (0.21) is taken as a $\varphi_{D,0}^{\mathrm{fl}}$ value. In this case, $\Phi_{\mathrm{RET}}$ is equal to 99.1%. Thus, it can be concluded that the sensing mechanism of the probe **NI**-**SP** is based on the inhibition of PET from the *N*-aryl receptor fragment to the NI chromophore upon interaction with an analyte, which in turn leads to an increase in RET efficiency between NI and SP from 91% to 99.7% (or 99.1%). Interestingly, such a low RET switching contrast explains the absence of pronounced changes in the emission intensity of the styrylpyridine unit and, hence, provides the possibility of dual-channel fluorescent detection of Hg(II) ions.

**Figure 4:**
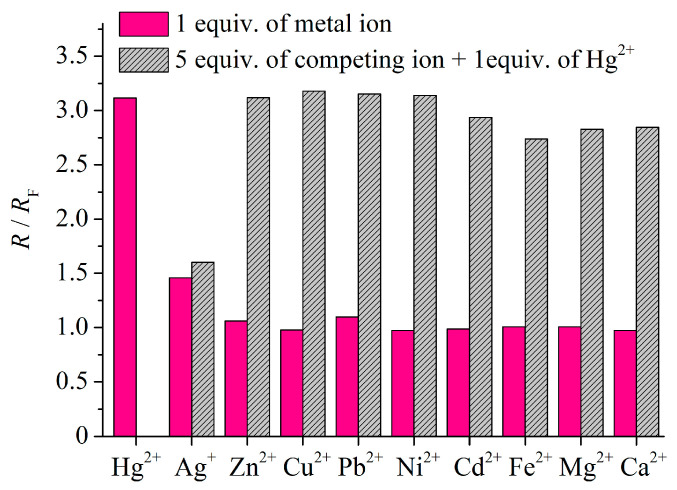
Selectivity of fluorescent response of compound **NI-SP** (10.0 μM) towards mercury (II) ions in water at pH 4.5 (acetate buffer, 0.1 M). The value *R*_F_ was calculated as the ratio of fluorescence intensity at 465 nm to that at 610 nm for the free ligand **NI-SP**, *R* is the same ratio but found after the addition of metal ions to the solution of **NI-SP**. Excitation wavelength is 375 nm.

We next studied the selectivity of the ratiometric response of the sensor **NI**-**SP**. The addition of 1 equiv. of Hg^2+^ induced an increase in the ratio of emission intensities at 465 and 610 nm, whereas no significant changes were observed in the presence of 1–5 equiv. of other metal ions except Ag^+^ (Figure 4 and Figures S4–S13). The competition experiments were also carried out for **NI-SP** (Figure 4). In a typical procedure, the fluorescence spectrum was collected before and after the addition of 5 equiv. of other metal ions into the solution of **NI-SP** (10.0 μM) containing 1 eq. of mercury (II) perchlorate. As can be seen, the presence of Zn^2+^, Cu^2+^, Pb^2+^, Ni^2+^, Cd^2+^, Fe^2+^, Mg^2+^ or Ca^2+^ did not appear to have any pronounced signal interference; however, in the case of Ag^+^, the ratio *R* was decreased. This result indicates that coordination of **NI**-**SP** with the silver cations is also possible and the concentration of Hg^2+^ cannot be quantified in the presence of Ag^+^. It could be supposed that Hg(II) bound with the A15C5 receptor is displaced by the silver cation upon the addition of AgClO_4_, the forming complex of **NI**-**SP** with Ag^+^ being characterized by a lower *R* value. Complexation with Ag^+^ was also observed for monochromophoric naphthalimides bearing the A15C5 group. Thus, compound **NI2** demonstrated a rather high selectivity for Ag^+^ over Hg^2+^ at pH 7.4 [40], whereas both Hg^2+^ and Ag^+^ cations were capable of binding with the A15C5 macrocycle in an acidic medium (pH 6.0, acetate buffer, see the data for the A15C5-containing naphthalimide derivative with a close to **NI2** structure [32]). In the latter case, the initially Hg^2+^-enhanced fluorescence of the naphthalimide unit was quenched in a similar fashion when competing silver cations were added to the solution.

### 3.4. Characteristics of Intracellular Accumulation, Localization and Retention of NI-SP

**Figure 5:**
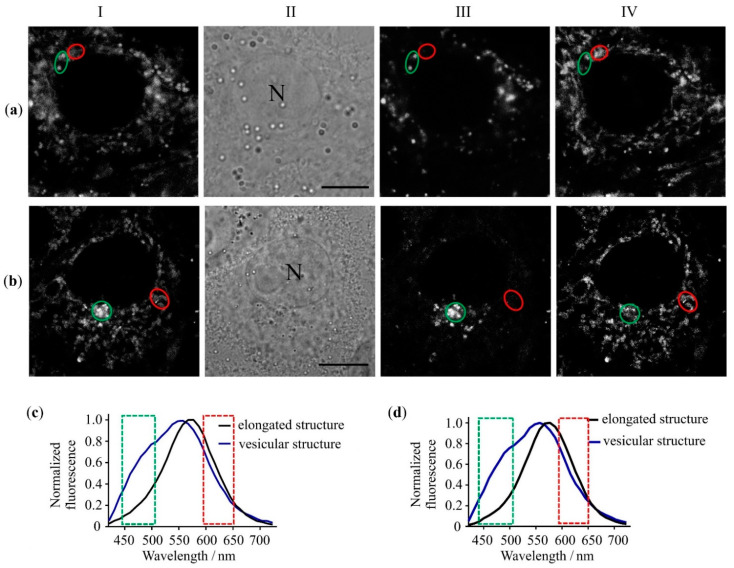
Confocal fluorescence images (**a**,**b**) and fluorescence spectra (**c**,**d**) of **NI-SP** in A549 (**a**,**c**) and HEK293 (**b**,**d**) cells. (**a**,**b**) Typical intracellular distributions (columns I, III and IV) of **NI-SP** in the 440–710 nm (column *I*), 440–510 nm (column III) and 590–650 nm (column IV) ranges. Transmitted-light images of cells are shown in column II. Bar represents 10 µm. «N» marks a nucleus. Cells were incubated with **NI-SP** (5 µM) for 30 min. (**c**,**d**). Typical intracellular fluorescence spectra of **NI-SP** in elongated and vesicular structures of A549 cells (**c**) and HEK293cells (**d**). Presented spectra were measured in cellular structures marked with green and red ovals rows (**a**,**b**). Spectra were excited at *λ*_ex_ = 405 nm. Green and red rectangles in panels *c*,*d* mark spectral regions, which were used to measure images shown in columns III and IV, respectively.

Interactions of **NI-SP** with cells of various histogenesis were studied to evaluate the applicability of **NI-SP** for the fluorescent imaging of Hg^2+^ in living cells. As shown with confocal laser scanning microscopy (CLSM) (Figure 5), **NI-SP** efficiently penetrates and accumulates in the cytoplasm of human lung adenocarcinoma A549 cells (cancer cells) and human embryonic kidney 293 cells (HEK293, normal cells). Compound **NI-SP** accumulates predominantly in vesicular structures of a submicron size and in elongated structures that form a complex network. Intracellular normalized fluorescence spectra of **NI-SP** excited at 405 nm were found to differ markedly in the vesicular and elongated cellular structures (Figure 5c). These spectral differences were observed in both types of the studied cells. The characteristic feature of the **NI-SP** fluorescence spectrum in vesicles is a prominent shoulder in the 440–510 nm range, which can be assigned to the increased contribution of donor moiety fluorescence. Accordingly, the confocal images recorded in the 440–510 nm range demonstrate the predominant vesicular distribution of **NI-SP**, while the images recorded in the 590–650 nm range show the localization of **NI-SP** in both vesicular and elongated cellular structures. Since the main features of **NI-SP** interactions with the A549 and HEK293 cells were found to be similar, further studies were continued using the A549 cells only.

Vesicular and elongated cellular structures, where **NI-SP** accumulates, were identified using vital fluorescent probes for cellular organelles. It was concluded that intracellular vesicles with **NI-SP** differ from lipid droplets (Figure S15). Most of these vesicles are lysosomes as it follows from the co-localization of **NI-SP** and LysoTracker Red (LTrR), the fluorescent probe for lysosomes (Figure S14). The co-localization coefficient of LTrR and **NI-SP** was calculated using the special function of the ImageJ program and found to be 0.7 ± 0.4. Elongated cellular structures with **NI-SP** were identified as mitochondria in the course of the co-localization analysis with Rhodamine 6G (Rh6G), the fluorescent probe for mitochondria (Figure S16). The corresponding co-localization coefficient was 0.9 ± 0.3.

To verify the probable accumulation of **NI-SP** in the endoplasmic reticulum (ER), ER-Tracker Green (BODIPY FL Glibenclamide, ERTG) was used. Cells were incubated with **NI-SP** (10 μM for 20 min, 37 °C) in the medium, then cells were washed twice with Hanks’ solution and incubated with ER-Tracker Green (BODIPY FL Glibenclamide); (1 μM, 15 min, 37 °C) in Hanks’ solution and studied using CLSM. The fluorescence of dyes was excited at 488 nm and imaged within the 500–710 nm range using the “lambda scan” (spectral) regime of the confocal microscope. The spectral unmixing procedure was used to deconvolve the measured intracellular spectra into the signals of NI-SP and ERTG (Figure S17). A web-like pattern of ER distribution revealed with ERTG does not coincide with the intracellular distribution of **NI-SP**, indicating the absence of **NI-SP** accumulation in the ER.

Analysis of the kinetics of the cellular uptake of **NI-SP** showed that saturation of the intracellular accumulation of **NI-SP** occurred after 1 h of the incubation of A549 cells with **NI-SP** (Figure S18). The half-accumulation time was 16 ± 3 min. The kinetics of **NI-SP** accumulation in lysosomes and mitochondria are different: very fast in lysosomes (Figure S18c) and slower in mitochondria (Figure S18d). The concentration dependence of the intracellular accumulation of **NI-SP** has a tendency to saturation at the **NI-SP** concentration higher than 5 µM (Figure S18b). The 50% level of intracellular accumulation is achieved at 4.6 ± 1.2 µM. At a low concentration (~1 µM), **NI-SP** accumulates predominantly in lysosomes (Figure S18e), while mixed accumulation in lysosomes and mitochondria is observed at the **NI-SP** concentration of 2.5 µM and higher (Figure S18f,g). Probe **NI-SP** is characterized by long retention in A549 cells (Figure S18a); 50% efflux of **NI-SP** from A549 cells occurs after 2.4 ± 0.1 h. Efflux of **NI-SP** is characterized by the accelerated release of **NI-SP** from mitochondria and its longer retention in lysosomes (Figure S18h–j).

### 3.5. Cellular Imaging of Hg^2+^ with Probe NI-SP

**Figure 6:**
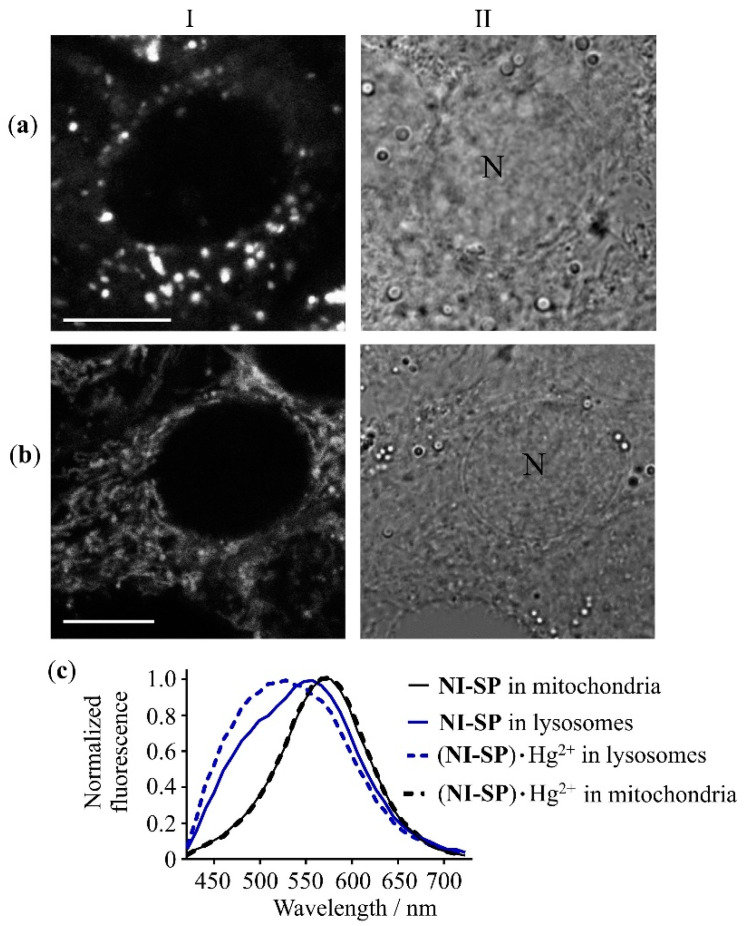
Confocal fluorescence images (**a**,**b**) and fluorescence spectra (**c**) of (**NI-SP**)∙Hg^2+^ in A549 cells at *λ*_ex_ = 405 nm. (**a**,**b**) Cells were pre-incubated with 20 μM of Hg(ClO_4_)_2_ for 20 min, washed twice with Hanks’ solution, and incubated with 10 μM of **NI-SP** for 20 min. (I) Images of intracellular fluorescence were measured in the 440–510 (**a**) and 590–650 nm (**b**) spectral ranges. (II) Conventional light microscope images of the cells. Bar represents 10 μm. «N» marks a nucleus. (**c**) Typical normalized intracellular fluorescence spectra of (**NI-SP**)∙Hg^2+^ and **NI-SP** in lysosomes and mitochondria.

In cells pre-incubated with Hg(ClO_4_)_2_, the intensity of **NI-SP** fluorescence in lysosomes is enhanced and the shape of the fluorescence spectra excited at *λ*_ex_ = 405 nm is changed, indicating the formation of (**NI-SP**)·Hg^2+^ complexes (Figure 6). In the presence of Hg^2+^ ions, the fluorescence spectra of **NI-SP** (*λ*_ex_ = 405 nm) in lysosomes become wider and show a maximum at 520 nm (Figure 6c). The characteristic spectral feature of (**NI-SP**)·Hg^2+^ complexes in lysosomes at *λ*_ex_ = 405 nm is an enhanced contribution of the donor moiety fluorescence in the 420–520 nm range (Figure 6c). In contrast, at *λ*_ex_ = 488 nm, fluorescence spectra of (**NI-SP**)·Hg^2+^ in lysosomes are changed only slightly as compared to the lysosomal spectra of **NI-SP** and demonstrate a small blue shift of the maximum from 572 to 567 nm (Figure S19c).

In mitochondria, the fluorescence spectra of **NI-SP** excited at 405 or 488 nm are not changed after the addition of Hg(ClO_4_)_2_ (Figure 6c and Figure S19c). The absence of changes in mitochondrial fluorescence spectra of **NI-SP** was observed at different incubation times of A549 cells with Hg^2+^ and **NI-SP** (5–60 min) in the wide range of concentrations of Hg(ClO_4_)_2_ (20–100 µM) and **NI-SP** (5–20 µM). One of the probable reasons is a basic pH (pH = 8.0) in mitochondria. We observed a similar phenomenon earlier with another fluorescent probe for Hg^2+^ ions [18].

**Figure 7:**
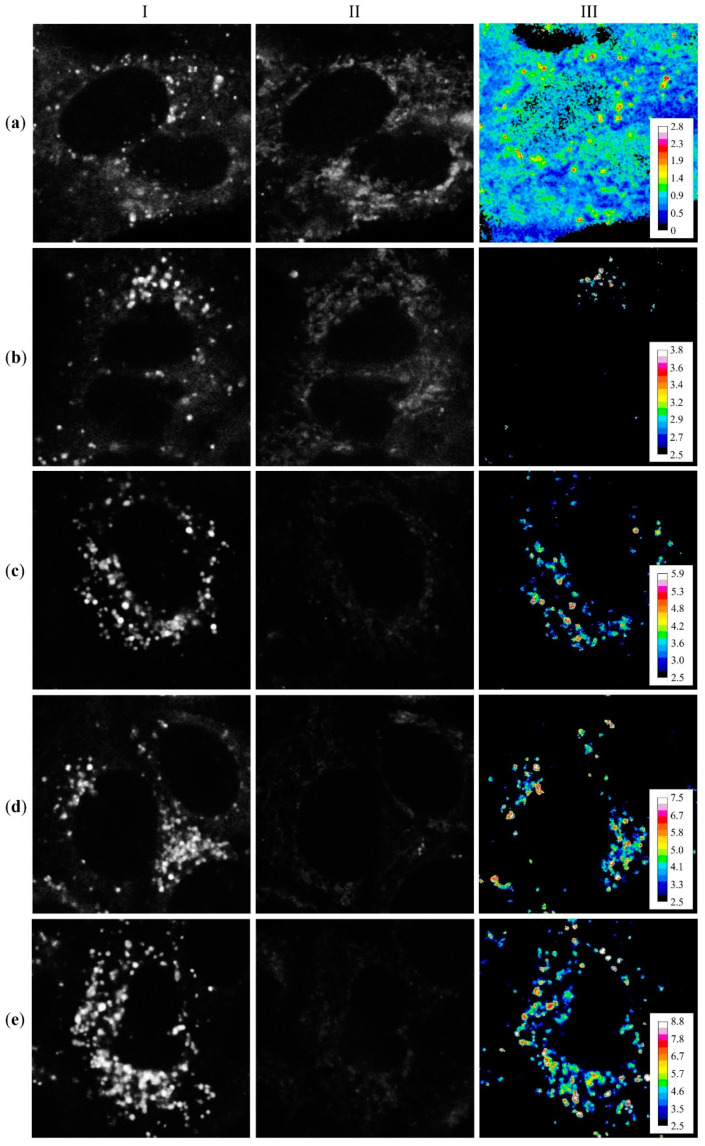
Fluorescent imaging of intracellular complexation of **NI-SP** with Hg^2+^. Fluorescence was excited at 405 nm and measured in the 440–510 nm (column I) and the 590–650 nm (column II) spectral ranges. Column III shows the ratio R′ of the fluorescence intensities *I*_440–510_/*I*_590–650_ calculated in every point of cell in the absence of Hg^2+^ ions row (**a**) and at 2.5 row (**b**), 5 row (**c**), 10 row (**d**) and 50 µM row (**e**) of Hg(ClO_4_)_2_. Intensity bar is the ratio R′ in every point of a cell. Cells A549 were pre-incubated with Hg(ClO_4_)_2_ for 20 min, washed twice with Hanks’ solution, and incubated with 10 μM of **NI-SP** for 20 min.

**Figure 8:**
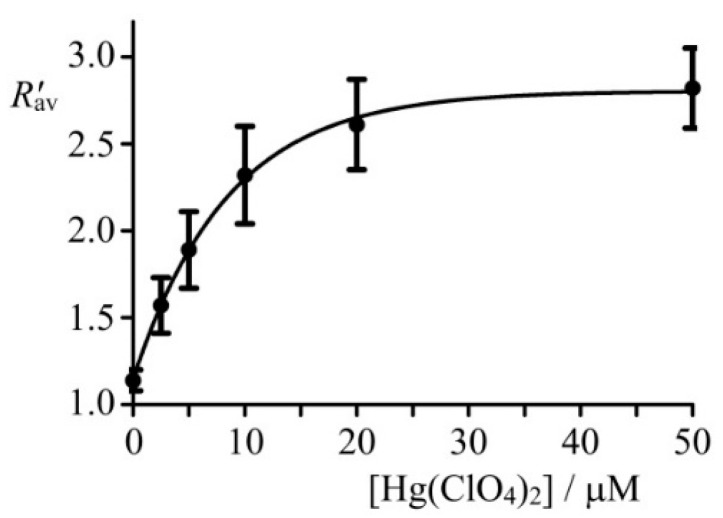
Dependence of the ratio $R\prime_{\mathrm{av}}$ on the concentration of mercury (II) perchlorate added to cells. Cells A549 were pre-incubated with different concentrations of Hg(ClO_4_)_2_ for 20 min, washed twice with Hanks’ solution and incubated with 10 μM of **NI-SP** for 20 min. Data were averaged over 20 cells in each measurement and presented as a mean ± SD.

The observed differences in the lysosomal fluorescence spectra of **NI-SP** and (**NI-SP**)·Hg^2+^ at *λ*_ex_ = 405 can be used for a ratiometric fluorescent detection of Hg^2+^ in cells. On the basis of integrated fluorescence intensities of **NI-SP** within cells measured simultaneously in the 440–510 and 590–650 nm spectral ranges at *λ*_ex_ = 405 nm (*I*_440–510_ and *I*_590650_, respectively), the images describing the intracellular distribution of the ratio *I*_440–510_/*I*_590650_ ($R^{\prime }$) can be obtained (Figure 7). This ratio is found to be sensitive to the presence of Hg^2+^ ions in cells increasing when the concentration of Hg(ClO_4_)_2_ added to cells rises (Figure 7 and Figure 8). Locally within cellular lysosomes, *R*′ varies from 1.4 to 2.5 in the absence of Hg^2+^ ions, and reaches 7.8–8.0 in some lysosomes at 50 µM of Hg(ClO_4_)_2_. Local $R^{\prime }$ values exceeding 2.8 clearly indicate the presence of Hg^2+^ in cells.

Dependence of the ratio $R^{\prime }$ averaged over confocal images of cells (${R^{\prime }}_{\mathrm{av}}$) on the concentration of Hg(ClO_4_)_2_ added to cells increases nonlinearly from 1.1 to 2.6–2.8 and shows saturation at the Hg(ClO_4_)_2_ concentration higher than 20 µM (Figure 8). The changes in ${R^{\prime }}_{\mathrm{av}}$ at 20–50 µM Hg(ClO_4_)_2_ are within the measurement accuracy, and, therefore, the upper limit of Hg^2+^ detection inside cells using **NI-SP** corresponds to ca. 20 µM of Hg^2+^ ions in the extracellular medium.

As shown previously [46], an average intracellular concentration of Hg^2+^ ions ${\left[{\mathrm{Hg}}^{2+}\right]}_{i,\mathrm{av}}$ can be estimated using Equation (2)

(2) $${\left[{\mathrm{Hg}}^{2+}\right]}_{i,\mathrm{av}}=K_{d}\cdot Q\cdot \frac{{R^{\prime }}_{\mathrm{av}}-{R^{\prime }}_{\mathrm{av},\min}}{{R^{\prime }}_{\mathrm{av},\max}-{R^{\prime }}_{\mathrm{av}}}\;$$

where $K_{d}$ is the dissociation constant of the (**NI-SP**)·Hg^2+^ complex calculated from lg *K* (6.89 ± 0.13, see Section 3.3) and equal to 0.13 ± 0.04 µM, ${R^{\prime }}_{\mathrm{av},\min}$ and ${R^{\prime }}_{\mathrm{av},\max}$ are the ${R^{\prime }}_{\mathrm{av}}$ values for the free ligand **NI-SP** (${R^{\prime }}_{\mathrm{av},\min}$ = 1.14 ± 0.06) and for the complex (**NI-SP**)·Hg^2+^ (${R^{\prime }}_{\mathrm{av},\max}$ = 2.82 ± 0.23) in cells found using the plot in Figure 8, *Q* is the ratio of the intracellular fluorescence intensity of the free ligand **NI-SP** to the intensity of (**NI-SP**)·Hg^2+^ in the 590–650 nm range (*Q* = 1.81 ± 0.04). Thus, at the extracellular Hg^2+^ concentrations of 2.5, 5.0, 10.0 and 20.0 µM the ${\left[{\mathrm{Hg}}^{2+}\right]}_{i,\mathrm{av}}$ values were 0.08, 0.19, 0.56 and 1.65 µM, respectively. The dependence of ${R^{\prime }}_{\mathrm{av}}$ on ${\left[{\mathrm{Hg}}^{2+}\right]}_{i,\mathrm{av}}$ in the 0–0.19 µM range demonstrated a good linearity with a correlation coefficient of 0.99 (Figure S20). Following formalism presented elsewhere [47], the detection limit for Hg^2+^ in cells ($C_{\mathrm{DL}}$) can be estimated from the slope of this linear dependence (r) and the standard deviation of the ratio ${R^{\prime }}_{\mathrm{av}}$ (s), using Equation (3).

(3) $$C_{\mathrm{DL}}=\frac{3s}{r}$$

where the value $\;C_{\mathrm{DL}}$ was calculated to be 46 nM. It is comparable with $C_{\mathrm{DL}}$ values of other fluorescent chemosensors for the Hg(II) ion [18,31,48].

It should be noted that the calculations of ${\left[{\mathrm{Hg}}^{2+}\right]}_{i,\mathrm{av}}$ by Equation (2) only allow quantification of the equilibrium concentration of the «free» mercury (II) ions. If one needs to find the total amount of Hg(II) inside cells (including Hg(II) trapped in stable complexes with some species such as thiol compounds), some other spectroscopic techniques such as atomic absorption spectroscopy or ICP-MS analysis should be used. Regarding complexation with thiols, we have previously shown that the presence of cysteine affects the sensing of Hg^2+^ in a slightly acidic aqueous solution by the probe bearing the same azadithia-15-crown-5 ether receptor group as in the structure **NI-SP** (see details in [18]).

To demonstrate the applicability of **NI-SP** for the monitoring of intracellular changes in Hg^2+^ concentration we measured the kinetics of the intracellular accumulation of Hg^2+^. Cells were incubated with **NI-SP** (10 µM, 20 min), washed twice with Hanks’ solution and exposed to 10 µM Hg(ClO_4_)_2_. The response of **NI-SP** to Hg^2+^ that was measured during the first 30 min of cell incubation with Hg(ClO_4_)_2_ revealed a fast increase in Hg^2+^ concentration in lysosomes during the first 5 min of incubation and saturation of this process in 10–15 min (Figure S21).

In order to verify the selectivity of the intracellular response of **NI-SP** to Hg^2+^ ions, A549 cells pre-incubated with Cu^2+^ or Pb^2+^ ions in the concentration range of 2–1000 µM were further incubated with **NI-SP** (10 µM) and studied with CLSM. In the presence of Cu^2+^ and Pb^2+^ ions, intracellular distribution and intracellular fluorescence spectra of **NI-SP** did not change and, accordingly, $R^{\prime }$ values were not affected compared to control cells (Figure S22). These results correlate with the data for the inability of **NI-SP** to form complexes with Cu^2+^ and Pb^2+^ in an aqueous solution (see Figure 4 and Figures S7 and S8). In the presence of AgClO_4_ in the cell medium, the intracellular distribution and intracellular fluorescence spectra of **NI-SP** did not change (Figures S23a and S24) and, accordingly, $R^{\prime }$ values were not affected compared to control cells (Figure 7). Thus, ${R^{\prime }}_{\mathrm{av}}$ values for control cells and cells treated with 0.2 mM AgClO_4_ were equal to 1.03 ± 0.12 and 1.06 ± 0.07, respectively. Similarly, the presence of AgClO_4_ in the cell medium did not affect the spectral response of **NI-SP** to Hg^2+^ ions in cells (Figures S23b and S24), and ${R^{\prime }}_{\mathrm{av}}$ values were equal to 2.6 ± 0.3 and 2.6 ± 0.3 for cells treated with 20 μM Hg(ClO_4_)_2_ alone and with 20 μM Hg(ClO_4_)_2_ and 0.2 mM AgClO_4_, respectively. Taking into account that the extracellular and intracellular concentrations of chloride anion are about 100 mM and the solubility product of silver chloride is rather low (1.77 × 10^–10^), the concentration of Ag^+^ ions in cells at sub-millimolar concentrations of added AgClO_4_ seems to be below the lower detection limit of **NI-SP**. Thus, the spectral response of **NI-SP** to Hg^2+^ ions in cells is selective, and **NI-SP** is promising for the ratiometric detection of Hg^2+^ ions in living cells.

The extended study of the cellular properties of **NI-SP** showed that **NI-SP** is not toxic to cells at the concentration of 20 µM or less for 24 h incubation (Figure S25a), and concentrations of **NI-SP** used in the present study did not affect cell growth. As for Hg(ClO_4_)_2_, survival of the A549 cells decreased to 95–90% when cells were treated with Hg(ClO_4_)_2_ at 2.5–40 µM for 48 h (Figure S25b).

## 4. Conclusions

In summary, we demonstrated that the probe **NI-SP** can be used for fluorescence imaging and the determination of Hg^2+^ concentration in living cells. In an aqueous solution, compound **NI-SP** shows dual emission spectrum, in which the short wavelength band arising from the energy donor NI-based PET-fluorophore is cation-dependent, whereas the long wavelength peak of the acceptor SP fragment is not changed upon Hg^2+^ coordination, thereby providing the ability to register a ratiometric fluorescence response. Thus, the sensing mechanism of **NI-SP** is based on the interplay between PET and RET upon excitation.

In contrast to **NI-SP**, a previously published Hg^2+^ probe bearing bisstyryl dye and *N*-phenylazadithia-15-crown-5 ether receptor [18] responds to the Hg^2+^ ion in a different way. In this compound, RET efficiency was switched as a result of ICT (intramolecular charge transfer) absorption band shift induced by the complexation of Hg^2+^ with an energy acceptor chromophore attached to the receptor group. In respect of the sensor characteristics, we found that the combination of PET and RET interactions realized in the design of **NI-SP** leads to a more preferable optical response because, in this case, the analytical signal (i.e. the ratio *R* of emission intensities at two different wavelengths) is increased in the presence of a metal cation. For the bisstyryl dye-based probe, we observed an opposite effect (a decrease of *R*). It is well-known that in terms of practical use «OFF → ON» signaling is better than «ON → OFF» signaling as the former is characterized by a lower noise/signal ratio. We consider that the data presented herein together with the results of our previous paper [18] show an example of how the optical response of a fluorescent probe can be modulated by the choice of an appropriate pair of chromophores.

We also showed that (i) the **NI-SP** probe is applicable to quantifying Hg(II) ions in solution: the calibration plot of R versus equiv. Hg^2+^ (Figure 3b) can be used to calculate the concentration of Hg(II) in an aqueous solution at pH 4.5; (ii) **NI-SP** is applicable to estimate a concentration of Hg(II) ions, to which cells were exposed, on the basis of the **NI-SP** response to Hg^2+^ in cells (${R^{\prime }}_{\mathrm{av}}$ value) and the calibration plot presented in Figure 8; and (iii) **NI-SP** is applicable to estimating roughly the equilibrium concentration of the «free» Hg^2+^ ions in lysosomes using Equation (2).

Regarding the selectivity of complex formations, the probe **NI**-**SP** was found to sense Hg^2+^ in the presence of other bio-relevant metal cations both in water and in an intracellular medium. The quantitative detection of Hg^2+^ inside cells with **NI-SP** is possible in the 0.04–1.65 μM concentration range. The presented results allow us to conclude that compound **NI-SP** can find applications in the studies of the role and transformations of mercury (II) cation in complex biological systems.

## Figures and Tables

**Table 1:** Spectral characteristics of compounds **NI1**, **NI2**, **SP** and **NI-SP** in water.

Species	$\lambda_{\max}^{\mathrm{abs}}/\mathrm{nm}$	$\varepsilon_{\lambda }/M^{–1}\cdot {\mathrm{cm}}^{–1}$	$\lambda_{\max}^{\mathrm{fl}}$ $(\lambda_{\mathrm{ex}})/\mathrm{nm}$	$\varphi^{\mathrm{fl}}$
**NI1**	374	13.6	467 (375)	0.71
**NI2**	370	9.7	465 (375)	0.0073
**SP**	450	28.3	619 (455)	0.00039
**NI-SP**	382; 482	14.8; 18.0	461; 605 (375)	0.00094

## Data Availability

Not applicable.

## Supplementary Materials

The following supporting information can be downloaded at: https://www.mdpi.com/article/10.3390/bios12090770/s1, theoretical calculation of RET efficiency in (**NI-SP**)·Hg^2+^ complex, steady-state absorption and fluorescence spectra of **NI-SP** probe and model compounds in the presence and absence of metal cations (Figures S1–S13), experimental details and results concerning the studies on intracellular localization of **NI-SP** (Figures S14–S17), kinetics of intracellular accumulation and retention of **NI-SP** (Figure S18), interaction of **NI-SP** with Hg^2+^, Cu^2+^, Pb^2+^ and Ag^+^ ions in cells (Figures S19–S24), cell viability (Figure S25), synthesis and characterization of the compounds (Figures S26–S37).
