# Supplementary material for: Fluorescent RET-Based Chemosensor Bearing 1,8-Naphthalimide and Styrylpyridine Chromophores for Ratiometric Detection of Hg2+ and Its Bio-Application
Source: Biosensors (Basel). 2022 Sep 19;12(9):770. doi: 10.3390/bios12090770 (PMC9497167; doi:10.3390/bios12090770)
Supplement: Supplementary file 1 [file biosensors-12-00770-s001.zip › biosensors-1819694-supplementary.pdf]

Supplementary Information

Fluorescent RET-based chemosensor bearing 1,8-naphthalimide and styrylpyridine chromophores for ratiometric detection of Hg^{2+} and its bio-application

Pavel A. Panchenko, Anastasija V. Efremenko, Anna S. Polyakova,
Alexey V. Feofanov, Maria A. Ustimova, Yuri V. Fedorov, Olga A. Fedorova

CONTENTS

1. Synthesis of the compounds……………………………………...……...……….…..…S2
2. Theoretical calculation of RET efficiency in (**NI-SP**)·Hg^{2+} complex.……...…...….…………..S5
3. Steady-state absorption and emission spectra in aqueous solutions…….……….....…...……S6
4. Intracellular localization of **NI-SP**……....………………………….…….…………..…….S10
5. Kinetics of intracellular accumulation and retention of **NI-SP**…….………………...….....…….S14
6. Interactions of **NI-SP** with Hg^{2+} in cells: analysis at λ_{ex} = 488 nm….…………………..…….S15
7. Plot of the ratio R'_{av} *versus* increasing concentrations of Hg^{2+} in cells.……..……….…….S16
8. Kinetic data on intracellular accumulation of Hg^{2+}……………………...………………..S16
9. Interactions of **NI-SP** with Cu^{2+}, Pb^{2+} and Ag^{+} in living cells...………………..…….…….S17
10. Cytotoxicity of **NI-SP** and $Hg(ClO_4)_2$…………………………....……………..……….S19
11. NMR and mass-spectra of the synthesized compounds……………………………………S20

1. Syntheses of the compounds

The reaction course and purity of the final products was followed by TLC on silica gel (DC-Alufolien Kieselgel 60 F_{254}, Merck). Flash chromatography was performed using a Biotage Isolera™ Prime system. Melting points were measured on Melt-temp melting point electrothermal apparatus and were uncorrected.

^{1}H and ^{13}C NMR spectra were recorded on an Avance 300 and Avance 400 spectrometers (Bruker). The measurements were performed in DMSO-d_6 and $CDCl_3$ solutions. The chemical shifts (given as δ) were determined with an accuracy of 0.01 ppm relative to the signals corresponding to the residual solvents and recalculated to the internal standard (TMS); the spin-spin coupling constants (J) were measured with an accuracy of 0.1 Hz. The assignment of ^{1}H and ^{13}C signals is based on 2D NMR experiments (HMBC, HSQC, ^{1}H COSY), which were performed using standard pulse sequences from the Bruker library.

LC-ESI-MS analyses were performed on a Finnigan LCQ Advantage mass spectrometer equipped with octopole ion-trap mass-analyzer, MS Surveyor pump, Surveyor auto sampler, Schmidlin-Lab nitrogen generator (Germany) and Finnigan X-Calibur 1.3 software for data collecting and processing. Isotope patterns were calculated with Molecular Weight Calculator, Version 6.37 (Matthew Monroe). Electron impact (EI) (70 eV) mass spectra were obtained from Finnigan Polaris Q instrument (ion-trap) in standard conditions.

Compound **4**. A mixture of 1-(3-bromopropyl)-4-methylpyridinium bromide (341 mg, 1.156 mmol) and sodium azide (188 mg, 2.892 mmol) in acetonitrile (5.0 mL) was refluxed for 16 h and then cooled to room temperature. The inorganic precipitate was filtered off, and the organic solution was evaporated in vacuo to afford product **4** as yellow oil. Yield 295 mg (99%). ^{1}H NMR (400.13 MHz, DMSO-d_6, 20°C, δ / ppm, J / Hz): 2.17 (m, 2H, CH_2(8)), 2.61 (s, 3H, Me), 3.46 (t, 2H, CH_2(9), ^{3}J = 6.8), 4.61 (t, 2H, CH_2(7), ^{3}J = 7.2), 8.0 (d, 2H, H(3), H(5), ^{3}J = 6.4), 8.94 (d, 2H, H(2), H(6), ^{3}J = 6.4). ^{13}C NMR (150.93 MHz, DMSO-d_6, 20°C, δ / ppm): 21.36 ($\underline{C}H_3$), 29.57 (C(8)), 47.54 (C(9)), 57.61 (C(7)), 128.32 (C(3), C(5)), 143.90 (C(2), C(6)), 158.91 (C(4)). ESI-MS in MeOH, m/z: calculated, 177.23; found, 177.17 ($[C_9H_{13}N_4]^+$). Found (%): C, 42.10; H, 5.15; N, 21.80. $C_9H_{13}BrN_4$ (MW 257.13). Calculated (%): C, 42.04; H, 5.10; N, 21.79.

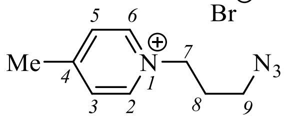

4

Compound **6**. A mixture of compound **4** (286 mg, 1.113 mmol), 4-(dimethyl-amino)benzaldehyde (216 mg, 1.450 mmol), piperidine (108 μL) in ethanol (6 mL) was refluxed for 8 h. Then, the solvent was evaporated and the residue was purified by column chromatography on SiO_2 using CH_2Cl_2–MeOH as an eluent to afford product **6** as red solid. Yield 243 mg (57 %).

M.p. 173–175°C. ^{1}H NMR (400.13 MHz, DMSO-d_6, 20°C, δ / ppm, J / Hz): 2.16 (m, 2H, CH_2(14)); 3.02 (s, 6H, NMe_2), 3.47 (t, 2H, CH_2(15), ^{3}J = 6.4), 4.48 (t, 2H, CH_2(13), ^{3}J = 6.9), 6.79 (d, 2H, H(11), H(13), ^{3}J = 8.9), 7.18 (d, 1H, H(7), ^{3}J = 16.5), 7.6 (d, 2H, H(10), H(14), ^{3}J = 8.9), 7.94 (d, 1H, H(8), ^{3}J = 16.5), 8.07 (d, 2H, H(3), H(5), ^{3}J = 6.4), 8.77 (d, 2H, H(2), H(6), ^{3}J = 6.4). ^{13}C NMR (150.93 MHz, DMSO-d_6, 20°C, δ / ppm): 29.51 (C(14)), 39.30 (NMe_2), 47.63 (C(15)), 56.81 (C(13)), 111.94 (C(11), C(13)), 117.10 (C(7)), 122.20 (C(9)), 122.38 (C(5), C(3)), 130.21 (C(10), C(14)), 142.30 (C(8)), 143.65 (C(2), C(6)), 151.93 (C(12)), 153.88 (C(4)). ESI-MS in MeOH, m/z: calculated 308.40; found 308.30 ($[C_{18}H_{22}N_5]^+$). Found (%): C, 55.72; H, 5.78; N, 18.06. $C_{18}H_{22}BrN_5$ (MW 388.20). Calculated (%): C, 55.68; H, 5.71; N, 18.04.

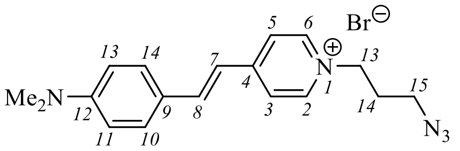

6

Compound **11**. To a stirring solution of compound **9** (87 mg, 0.151 mmol) in DMF (2.0 mL) propargyl alcohol (0.728 mmol, 42 μL) and K_2CO_3 (0.413 mmol, 57 mg) were added. The mixture was heated to 80°C and kept stirring at this temperature in argon atmosphere during 10 h. Then, the mixture was diluted with brine, and the precipitate was collected by filtration. The product was washed with distilled water, ethanol and dried in air. Yield 68 mg (78%). M.p. 233 – 234°C. ^{1}H NMR (400.13 MHz, DMSO-d_6, 20°C, δ / ppm, J / Hz): 2.72 – 2.80 (m, 4H, H(16), H(23)), 2.84 – 2.90 (m, 4H, H(17), H(22)), 3.58 – 3.67 (m, 8H, CH_2(18), CH_2(19), CH_2(20), CH_2(21)), 3.70 – 3.75 (m, 4H, CH_2(15), CH_2(24)), 3.76 (t, 1H, C≡CH, ^{3}J = 2.2), 5.24 (d, 2H, C$\underline{H_2}$C≡CH, ^{4}J = 2.1), 6.69 (d, 2H, H(11), H(13), ^{3}J = 8.9), 7.1 (d, 2H, H(10), H(14), ^{3}J = 8.9), 7.42 (d, 1H, H(3), ^{3}J = 8.3), 7.85 – 7.90 (m, 1H, H(6)), 8.48 (d, 1H, H(2), ^{3}J = 8.3), 8.52 (d, 1H, H(7), ^{3}J = 7.4), 8.58 (d, 1H, H(5), ^{3}J = 8.5). ^{13}C NMR (150.93 MHz, ДМСО-d_6, 25°C, δ / ppm): 29.01 (C(16), C(23)), 30.56 (C(17), C(22)), 51.36 (C(15), C(24)), 56.86 ($\underline{C}H_2$C≡CH), 70.03 (C(19), C(20)), 72.99 (C(18), C(21)), 78.16 (CH_2C≡$\underline{C}$H), 79.47 ($CH_2\underline{C}$≡CH), 107.65 (C(3)), 111.26 (C(11), C(13)), 115.61 (C(1)), 122.86 (C(4a)), 123.01 (C(8)), 124.05 (C(9)), 126.70 (C(6)), 128.16 (C(5)), 129.03 (C(8a)), 129.72 (C(10), C(14)), 131.18 (C(7)), 132.79 (C(2)), 146.31 C(12)), 157.98 (C(4)), 163.45 (C(8b)), 164.10 (C(8c)). EI-MS, m/z (I, %): 576 ($[M]^+$) (29), 428 (27), 415 (53), 381 (37), 369 (44), 355 (100), 342 (23), 316 (26), 287 (30), 234 (48), 151 (27). Found (%): C, 64.49; H, 5.63; N, 4.83; O, 13.91; S, 11.09. $C_{31}H_{32}N_2O_5S_2$ (MW 576.73). Calculated (%): C, 64.56; H, 5.59; N, 4.86; O, 13.87; S, 11.12.

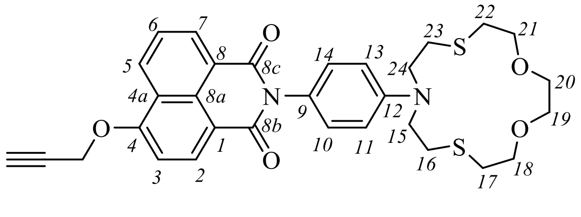

11

Compound **NI-SP**. A mixture of naphthalimide **11** (40 mg, 0.069 mmol), styrylpyridine **6** (27 mg, 0.070 mmol), DIPEA (25 μL, 0.144 mmol), CuI (5mg, 0.026 mmol) and DMF (3.0 mL) was stirred at 80°C in argon atmosphere during 12 h. The solvent was removed under reduced pressure, and the residue was purified using flash-chromatography (Al_2O_3, gradient mixture CH_2Cl_2 – MeOH) to give 30 mg of **NI-SP** (yield 45%). M.p. 238 – 239°C. ^{1}H NMR (400.13 MHz, DMSO-d_6, 20°C, δ / ppm, J / Hz): 1.13 – 1.25 (m, 2H, CH_2(29)), 2.73 – 2.80 (m, 4H, CH_2(16), CH_2(23)), 2.82 – 2.92 (m, 4H, CH_2(17), CH_2(22)), 3.02 (s, 6H, NMe_2), 3.48 – 3.53 (m, 2H, CH_2(30)), 3.55 – 3.68 (m, 8H, CH_2(18), CH_2(19), CH_2(20), CH_2(21)), 3.68 – 3.76 (m, 4H, CH_2(15), CH_2(24)), 4.49 – 4.58 (m, 2H, CH_2(28)), 5.54 (c, 2H, CH_2(25)), 6.68 (d, 2H, H(11), H(13), ^3J = 8.4), 6.67 (d, 2H, H(11'), H(13'), ^3J = 8.3), 7.05 – 7.17 (m, 3H, H(10), H(14) H(7')), 7.49 – 7.59 (m, 3H, H(10'), H(14'), H(3)), 7.80 – 7.93 (m, 2H, H(6), H(8')), 8.03 (d, 2H, H(3'), H(5'), ^3J = 6.1), 8.39 – 8.46 (m, 2H, H(2), H(27)), 8.47 – 8.58 (m, 2H, H(7), H(5)), 8.74 (d, 2H, H(2'), H(6'), ^3J = 6.1). ^{13}C NMR (150.93 MHz, DMSO-d_6, 25°C, δ / ppm): 29.49 (C(16), (C(23)), 29.87 (C(29)), 31.08 (C(17), C(22)), 39.20(NMe$_2$), 47.28 (C(30)), 51.80 (C(15), C(24)), 57.32 (C(28)), 62.95 (C(25)), 70.18 (C(19), C(20)), 70.60 (C(18), C(21)), 107.90 (C(3)), 111.74 (C(11), C(13)), 112.49 (C(11'), C(13')), 115.71 (C(1)), 117.45 (C(7')), 122.61 (C(9')), 122.83 (C(3'), C(5')), 123.12 (C(4a)), 123.51 (C(8)), 124.57 (C(9)), 125.38 (C(27)), 126.94 (C(6)), 128.81 (C(5)), 129.50 (C(8a)), 130.20 (C(10), C(14)), 130.67 (C(10'), C(14')), 131.66 (C(7)), 133.52 (C(2)), 141.71 (C(26)), 142.75 (C (8')), 144.01 (C(2'), C(6')), 146.74 (C(12)), 152.43 (C(12')), 154.31 (C(4')), 159.40 (C(4)), 163.92 (C(8b)), 164.48 (C(8c)). ESI-MS in MeCN, m/z: calculated 884.36; found 884.74 ($[C_{49}H_{54}N_7O_5S_2]^+$). Found (%): C, 60.98; H, 5.69; N, 10.18. $C_{49}H_{54}BrN_7O_5S_2$ (MW 966.04). Calculated (%): C, 60.92; H, 5.74; N, 10.15.

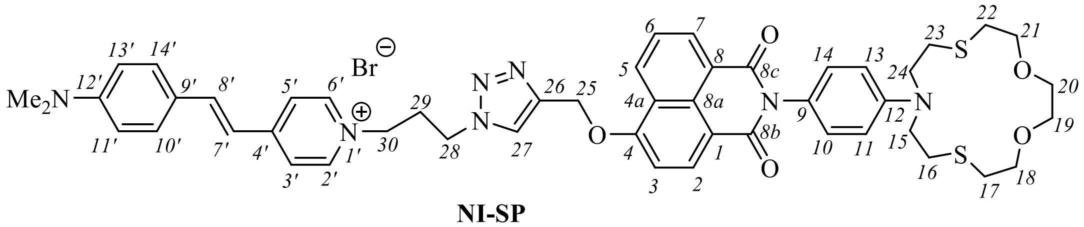

NI-SP

2. Theoretical calculation of RET efficiency in (NI-SP)·Hg^{2+} complex

Resonance energy transfer efficiency Φ_{RET} in the (**NI-SP**)·Hg^{2+} complex was calculated by the Equation (S1)[1]:

$$\Phi_{\mathrm{RET}} = \frac{R_0^6}{R_0^6 + r^6} \tag{S1}$$

where r is the distance between the donor and the acceptor and R_0 is the critical Förster radius at which 50% of the excited donors deactivate through the RET pathway (*i.e.* $\Phi_{\mathrm{RET}} = 0.5$). The value r was found from the optimized ground state geometry of the free ligand **NI-SP** (r = 12.3 Å, see the manuscript text), and R_0 (Å) was calculated according to the Equation (S2)[1]:

$$R_0^6 = 8.79{\cdot}10^{-5}\left[\kappa^2 \cdot n^{-4} \cdot \varphi_{D,0}^{\mathrm{fl}} \cdot J(\lambda)\right] \tag{S2}$$

where κ^2 is an orientation factor which depends on the mutual disposition of the donor and acceptor transition dipole moments, $\varphi_{D,0}^{\mathrm{fl}}$ is the fluorescence quantum yield of the donor in the absence of the acceptor, n is the refractive index of the solvent (water), N_A is Avogadro constant and $J(\lambda)$ is the overlap integral which defines the extent of overlap between the emission spectrum of the donor ($F_D(\lambda)$) and the absorption spectrum of the acceptor ($\varepsilon_A(\lambda)$, see Fig.S1 for the graphical representation of this overlap). The value $J(\lambda)$ was calculated according the Equation (S3)[1]:

$$J(\lambda) = \int_0^\infty F_{\mathrm{D}}(\lambda)\varepsilon_{\mathrm{A}}(\lambda)\lambda^4 d\lambda \Big/ \int_0^\infty F_{\mathrm{D}}(\lambda) d\lambda \tag{S3}$$

As a value $\varphi_{D,0}^{\mathrm{fl}}$, we used fluorescence quantum yield of the compound **NI1** (0.71, Table 1). The overlap integral $J(\lambda)$ was found to be $9.14{\cdot}10^{14}$ $M^{-1}{\cdot}nm^4{\cdot}cm^{-1}$, and the critical distance R_0 for the RET pair – 47.9 Å. According to the equation (S1), this corresponds to 99.97% of the photoexcited energy donor NI chromophores in the complex (**NI-SP**)·Hg^{2+} relaxing through the RET pathway.

---

[1]see J. R. Lakowicz, Principles of Fluorescence Spectroscopy, Springer, New York, USA, 2006.

3. Steady-state absorption and emission spectra in aqueous solutions

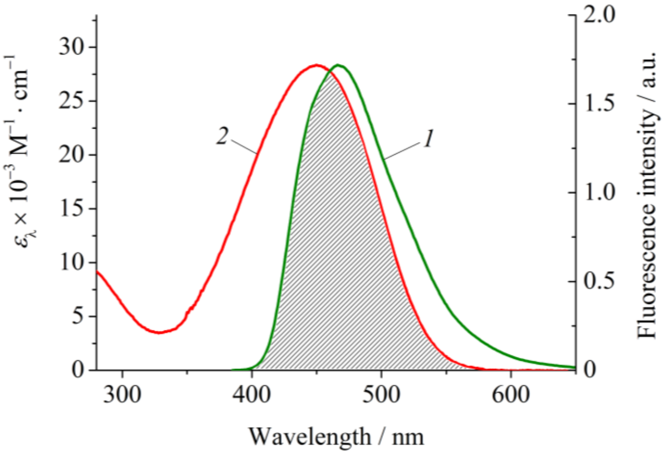

Figure S1. Overlap between emission spectrum of **NI1** (*1*) and absorption spectrum of **SP** (*2*) in water. Excitation wavelength is 375 nm. Concentrations of all compounds are 10.0 μM.

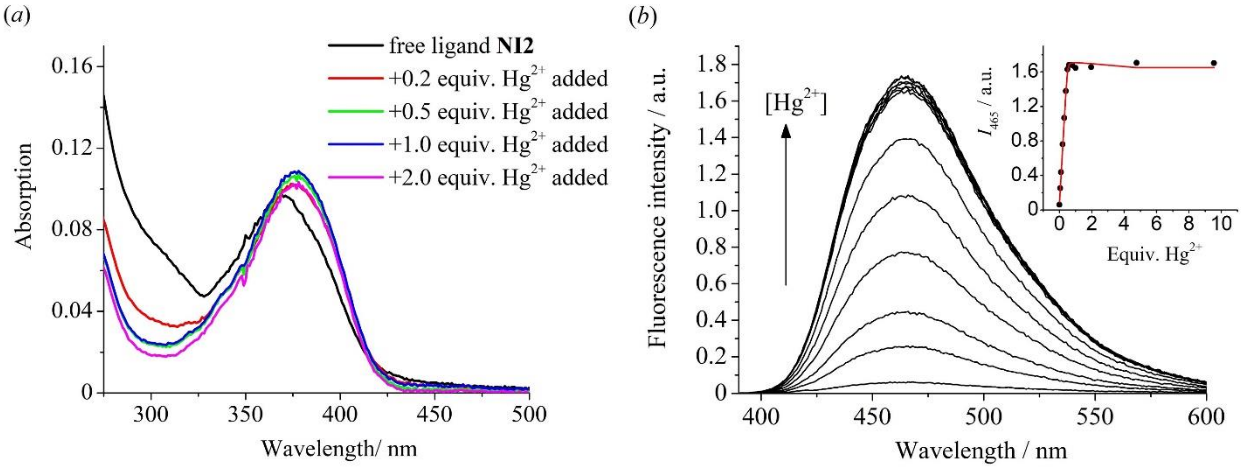

Figure S2. Changes in the absorption (*a*) and fluorescence (*b*) spectra of compound **NI2** (10.0 μM) upon addition of $Hg(ClO_4)_2$ at pH 4.5 (acetate buffer, 0.1 M). Excitation wavelength is 375 nm. The insert in panel (*b*) shows the fluorescence intensity at 465 nm (I_{465}) *versus* equivalents of Hg^{2+} in the solution.

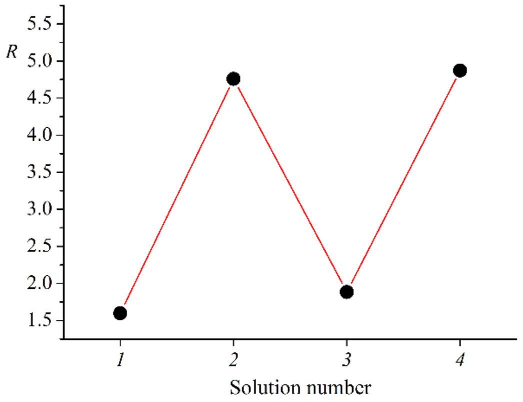

Figure S3. Ratio of fluorescence intensity at 465 nm to that at 610 nm (*R*) upon alternant addition of $Hg(ClO_4)_2$ and EDTA to the aqueous solution of probe **NI-SP** (10.0 μM) at pH 4.5 (0.1 M acetate buffer). Excitation wavelength 375 nm. *1* – free ligand **NI-SP**, *2* – 1 eq. Hg^{2+} added, *3* – 5 eq. EDTA added, *4* – 8 eq. Hg^{2+} added.

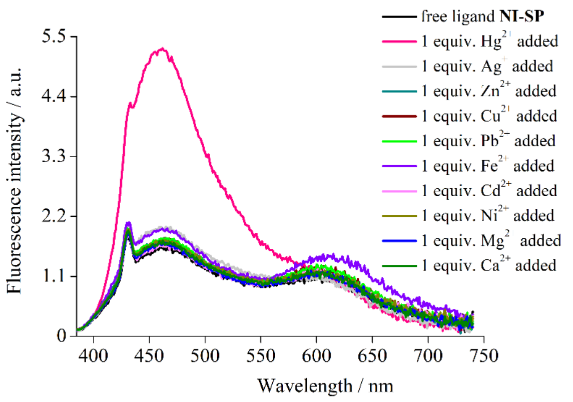

Figure S4. Fluorescence spectra of compound **NI2** (10.0 μM) in the presence and absence of 1 equiv. of various metal cations in water at pH 4.5 (acetate buffer, 0.1 M). Excitation wavelength is 375 nm.

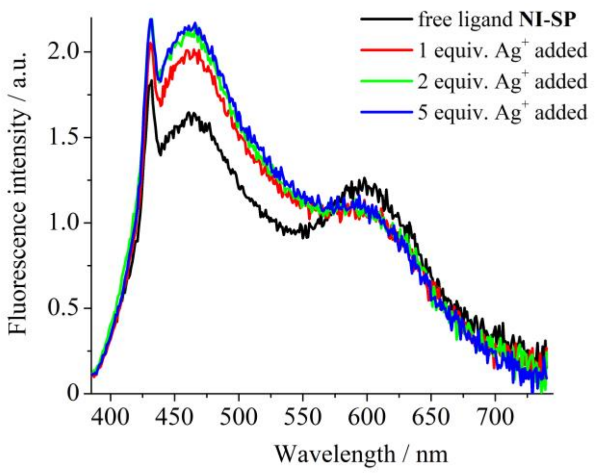

Figure S5. Fluorescence spectra of compound **NI-SP** (10.0 μM) upon gradual addition of $AgClO_4$ in water at pH 4.5 (acetate buffer, 0.1 M). Excitation wavelength is 375 nm.

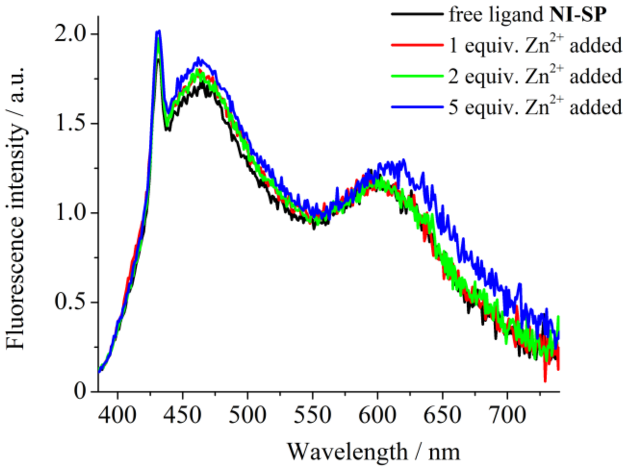

Figure S6. Fluorescence spectra of compound **NI-SP** (10.0 μM) upon gradual addition of $Zn(ClO_4)_2$ in water at pH 4.5 (acetate buffer, 0.1 M). Excitation wavelength is 375 nm.

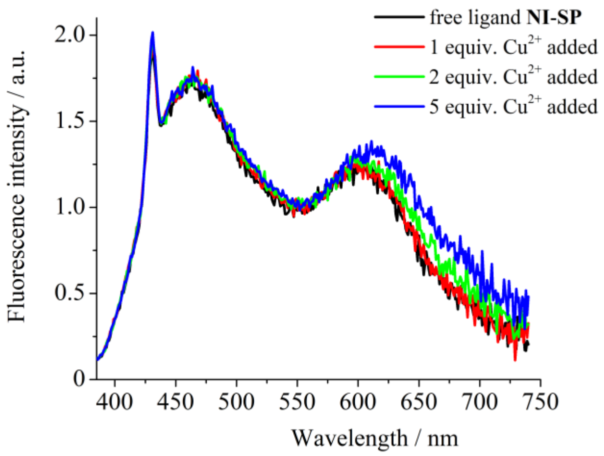

Figure S7. Fluorescence spectra of compound **NI-SP** (10.0 μM) upon gradual addition of $Cu(ClO_4)_2$ in water at pH 4.5 (acetate buffer, 0.1 M). Excitation wavelength is 375 nm.

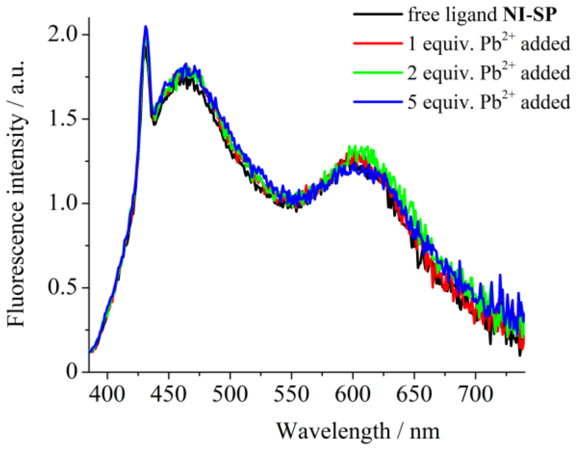

Figure S8. Fluorescence spectra of compound **NI-SP** (10.0 μM) upon gradual addition of $Pb(ClO_4)_2$ in water at pH 4.5 (acetate buffer, 0.1 M). Excitation wavelength is 375 nm.

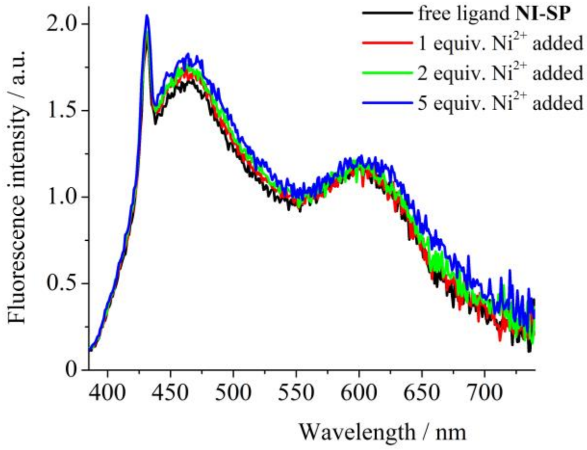

Figure S9. Fluorescence spectra of compound **NI-SP** (10.0 μM) upon gradual addition of $Ni(ClO_4)_2$ in water at pH 4.5 (acetate buffer, 0.1 M). Excitation wavelength is 375 nm.

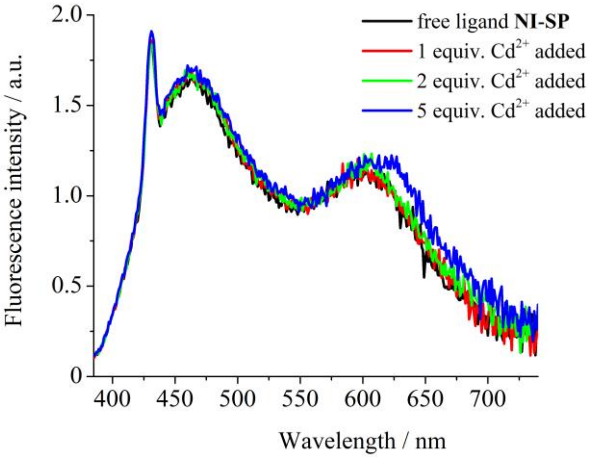

Figure S10. Fluorescence spectra of compound **NI-SP** (10.0 μM) upon gradual addition of $Cd(ClO_4)_2$ in water at pH 4.5 (acetate buffer, 0.1 M). Excitation wavelength is 375 nm.

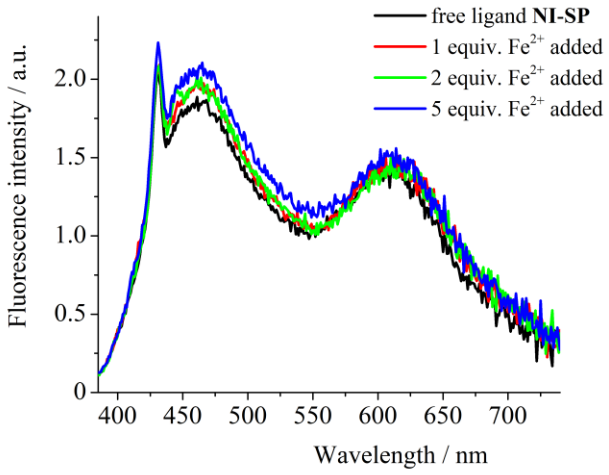

Figure S11. Fluorescence spectra of compound **NI-SP** (10.0 μM) upon gradual addition of $Fe(ClO_4)_2$ in water at pH 4.5 (acetate buffer, 0.1 M). Excitation wavelength is 375 nm.

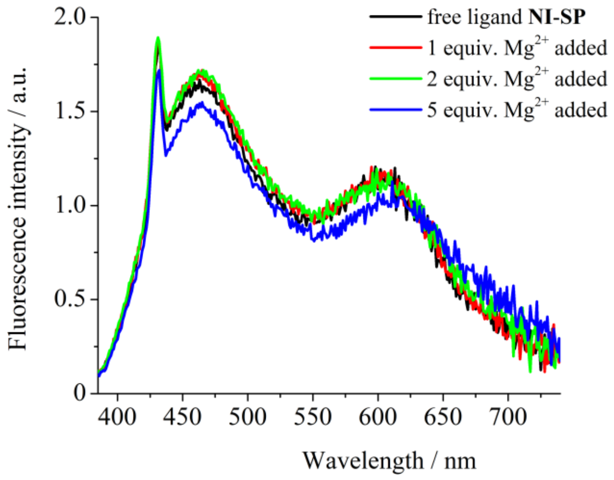

Figure S12. Fluorescence spectra of compound **NI-SP** (10.0 μM) upon gradual addition of $Mg(ClO_4)_2$ in water at pH 4.5 (acetate buffer, 0.1 M). Excitation wavelength is 375 nm.

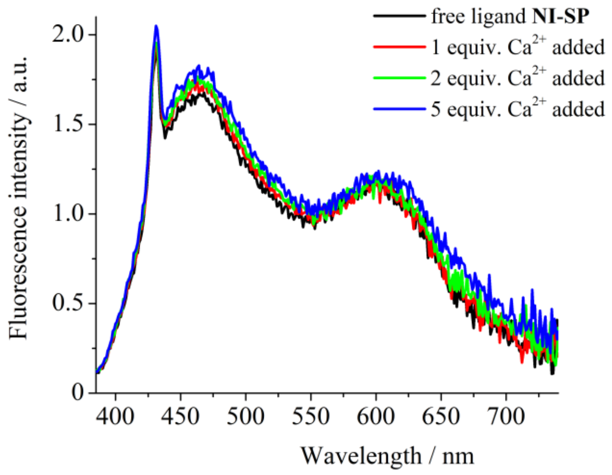

Figure S13. Fluorescence spectra of compound **NI-SP** (10.0 μM) upon gradual addition of $Ca(ClO_4)_2$ in water at pH 4.5 (acetate buffer, 0.1 M). Excitation wavelength is 375 nm.

4. Intracellular localization of NI-SP

To identify granular structures that accumulate **NI-SP**, LysoTracker Red (LtrR) was selected as a vital fluorescent of lysosomes, which fluoresces in the 560–750 nm range. In order to separate fluorescent signals of **NI-SP** and LtrR, fluorescence of **NI-SP** was excited at 405 nm and collected in the 440-510 nm spectral range, while emission of LtrR was registered in the 650–750 nm spectral range at the 543 nm excitation. As shown using CLSM, most of the intracellular granules with **NI-SP** are lysosomes (Fig. S14). Colocalization coefficient for **NI-SP** and LtrR (i.e. lysosomes), which was calculated using the Colocalization Threshold function of the ImageJ program (National Institute of Health, USA), is 0.74 ±0.5.

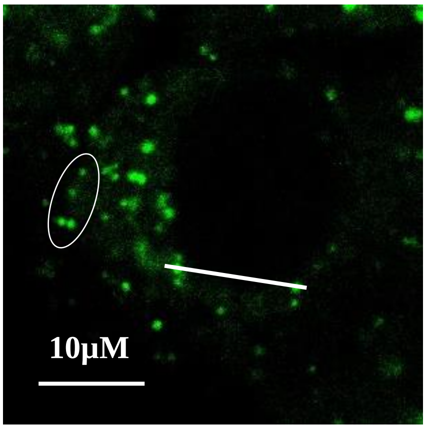

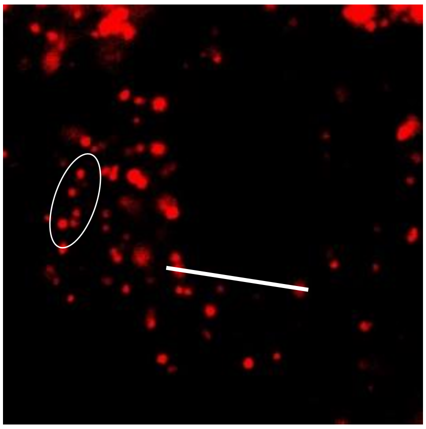

(a) (b)

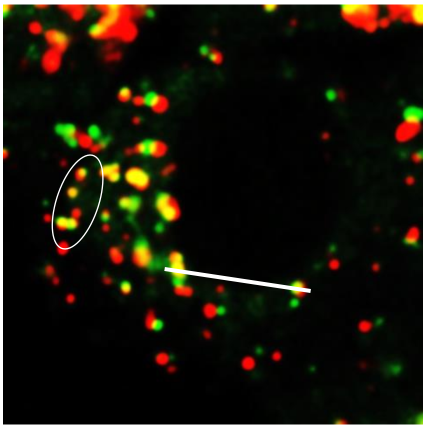

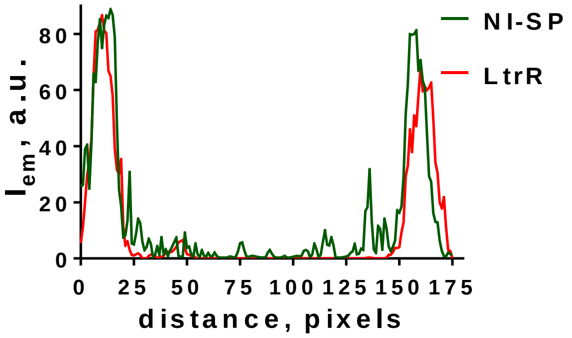

(c) (d)

Figure S14. Staining of lysosomes with LtrR in living A549 cells loaded with **NI-SP**. (**a,b**) Intracellular distributions of **NI-SP (a)** or LtrR **(b)** registered using CLSM. (**c**) Overlap of **(a)** and **(b)** showing intracellular distribution of **NI-SP** (green) or LtrR (red). Yellow color indicates colocalization of **NI-SP** and LtrR in lysosomes. Ovals highlight regions, where colocalization of **NI-SP** and LtrR is most obvious. (**d**) Distribution of fluorescence intensities of **NI-SP** and LtrR along a white line passing through two vesicles (see panels (**a**)–(**c**)). Close similarity of both intensity profiles confirms colocalization of **NI-SP** and LtrR in these vesicles. Bar represents 10 µm.

Comparing distribution of **NI-SP** fluorescence with the distribution of lipid droplets, which can be observed in transmitted-light images, it was concluded that **NI-SP** does not accumulate in lipid droplets, cellular organelles for storage of neutral lipids (Fig. S15).

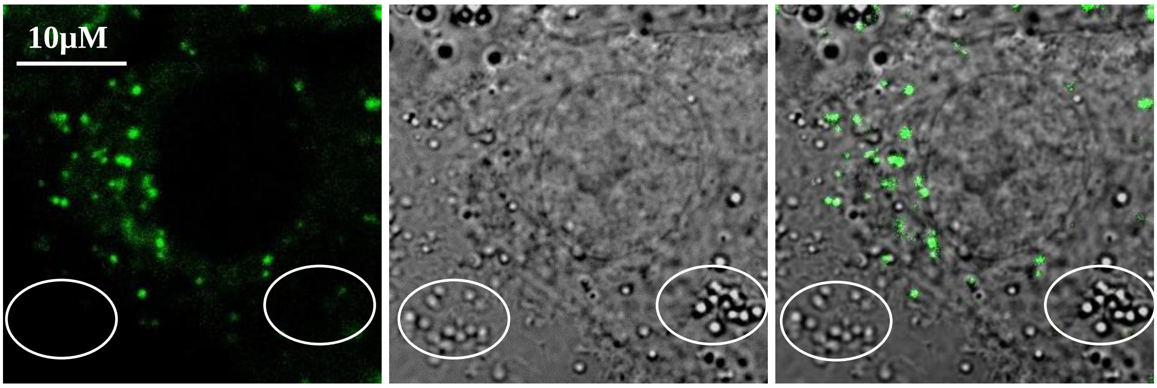

(a) (b) (c)

Figure S15. Accumulation sites of **NI-SP** in A549 cells are different from lipid droplets. (**a**) A confocal fluorescent image showing intracellular distribution of **NI-SP**. Bar represents 10 μm. (**b**) Transmitted light image of cells. Ovals mark two groups of lipid droplets situated at a cell periphery and observed as light-contrast granules. (**c**) Merged images (**a**) and (**b**) demonstrate absence of **NI-SP** accumulation in lipid droplets.

To check the accumulation of **NI-SP** in mitochondria, cells were incubated with 2 μM Rhodamine 6G (Rh6G; 10 min, 37 °C) after incubation with **NI-SP** (5 μM for 20 min, 37 °C) and studied using CLSM (Fig. S16). Fluorescence of Rh6G was excited at 543 nm and imaged within the 550–600 nm range. Fluorescence of **NI-SP** was excited at 405 nm, and imaged within the 600–700 nm range. Control cells were separately incubated with **NI-SP** or Rh6G and measured at the same conditions. The spectral crosstalk coefficient was 0.07 for **NI-SP** in the 550–600 nm range. For Rh6G, the spectral crosstalk coefficient was < 0.01 in the 600–700 nm range. Fluorescent CLSM images of intracellular distribution of **NI-SP** and Rh6G, which were corrected for the spectral crosstalk, demonstrate high level of colocalization of **NI-SP** with Rh6G in mitochondria (Fig. S16).

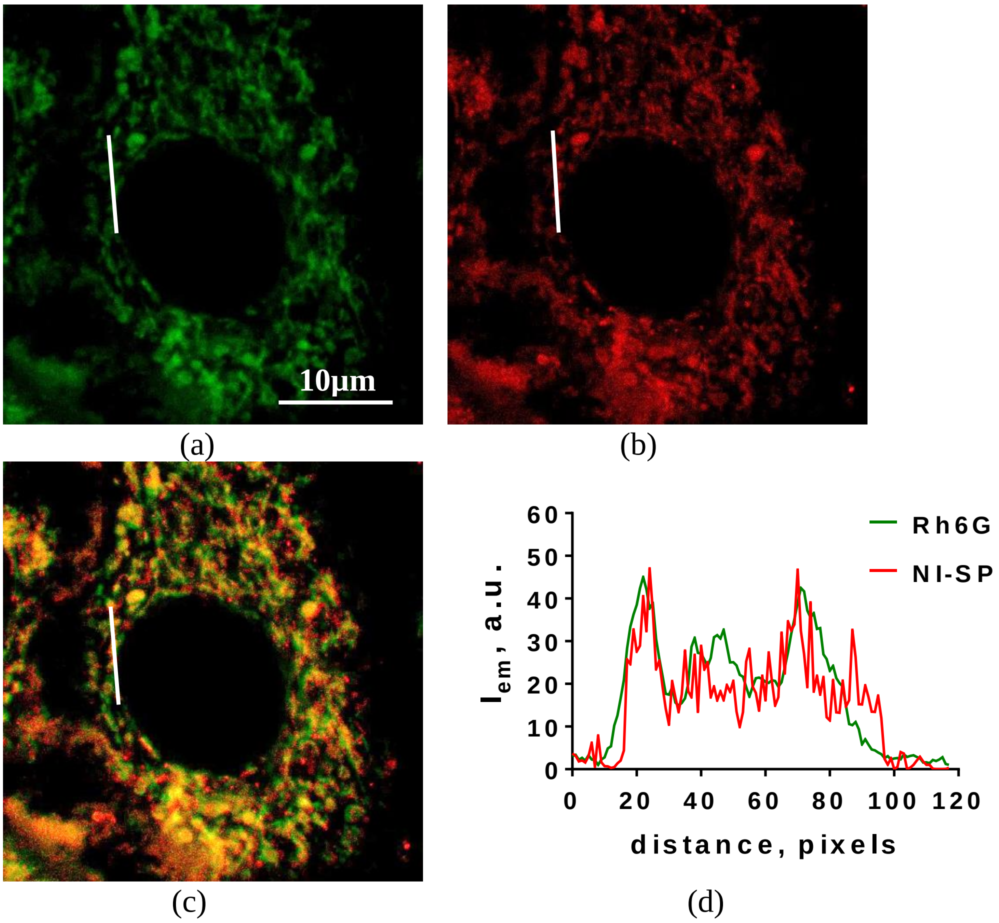

Figure S16. Identification of cellular organelles, which accumulate **NI-SP** in the A549 cells. (**a**, **b**) Intracellular distributions of Rhodamine 6G, a vital fluorescent probe of mitochondria **(a)** and **NI-SP** (**b**). Bar represents 10 µm. (**c**) Merged images (**a**) and (**b**). Yellow color indicates colocalization of **NI-SP** and Rh6G in mitochondria. (**d**) Distribution of fluorescence intensities of **NI-SP** and Rh6G along a white line (see panels (**a**)–(**c**)). Close similarity of both intensity profiles confirms colocalization of **NI-SP** and Rh6G in in mitochondria.

To verify probable accumulation of **NI-SP** in endoplasmic reticulum (ER), ER-Tracker Green (BODIPY FL Glibenclamide, ERTG) was used. Cells were incubated with **NI-SP** (10 µM for 20 min, 37 °C) in medium, then cells were washed twice with Hanks' solution and incubated with ER-Tracker Green (BODIPY FL Glibenclamide); (1 µM, 15 min, 37 °C) in Hanks' solution and studied using CLSM. Fluorescence of dyes was excited at 488 nm and imaged within the 500–710 nm range using "lambda scan" (spectral) regime of the confocal microscope. The spectral unmixing procedure was used to deconvolve the measured intracellular spectra into the signals of **NI-SP** and ERTG (Fig. S17). Web-like pattern of ER distribution revealed with ERTG does not coincide with the intracellular distribution of **NI-SP**, indicating absence of **NI-SP** accumulation in ER.

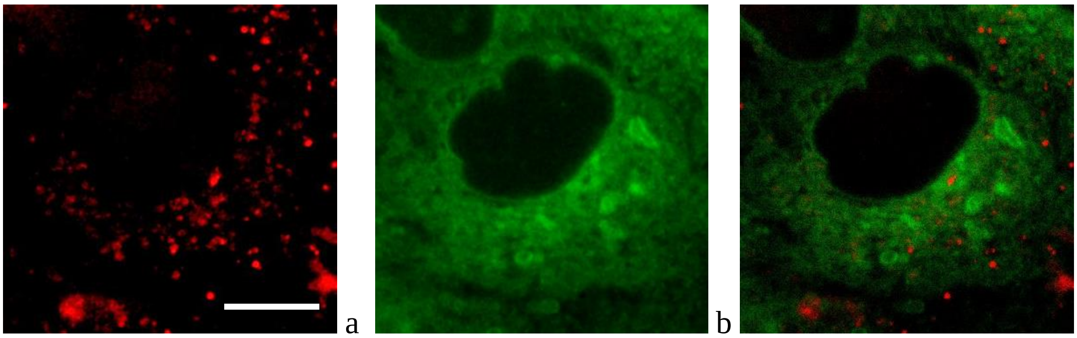

a b c

Figure S17. Identification of cellular organelles, which accumulate **NI-SP** in the A549 cells. (**a**, **b**) Intracellular distributions of NI-SP (a) and ERTG, a vital fluorescent probe of ER (**b**). Bar represents 10 μm. (**c**) Merged images (**a**) and (**b**). Cells were incubated with **NI-SP** (10 μM for 20 min, 37 °C) in medium, then cells were washed twice with Hanks' solution and incubated with ERTG (1 μM, 15 min, 37 °C) in Hanks' solution and studied using CLSM. Fluorescence of dyes was excited at 488 nm, imaged within the 500–710 nm range using a spectral regime and deconvolved into the components using a spectral unmixing procedure.

5. Kinetics of intracellular accumulation and retention of NI-SP

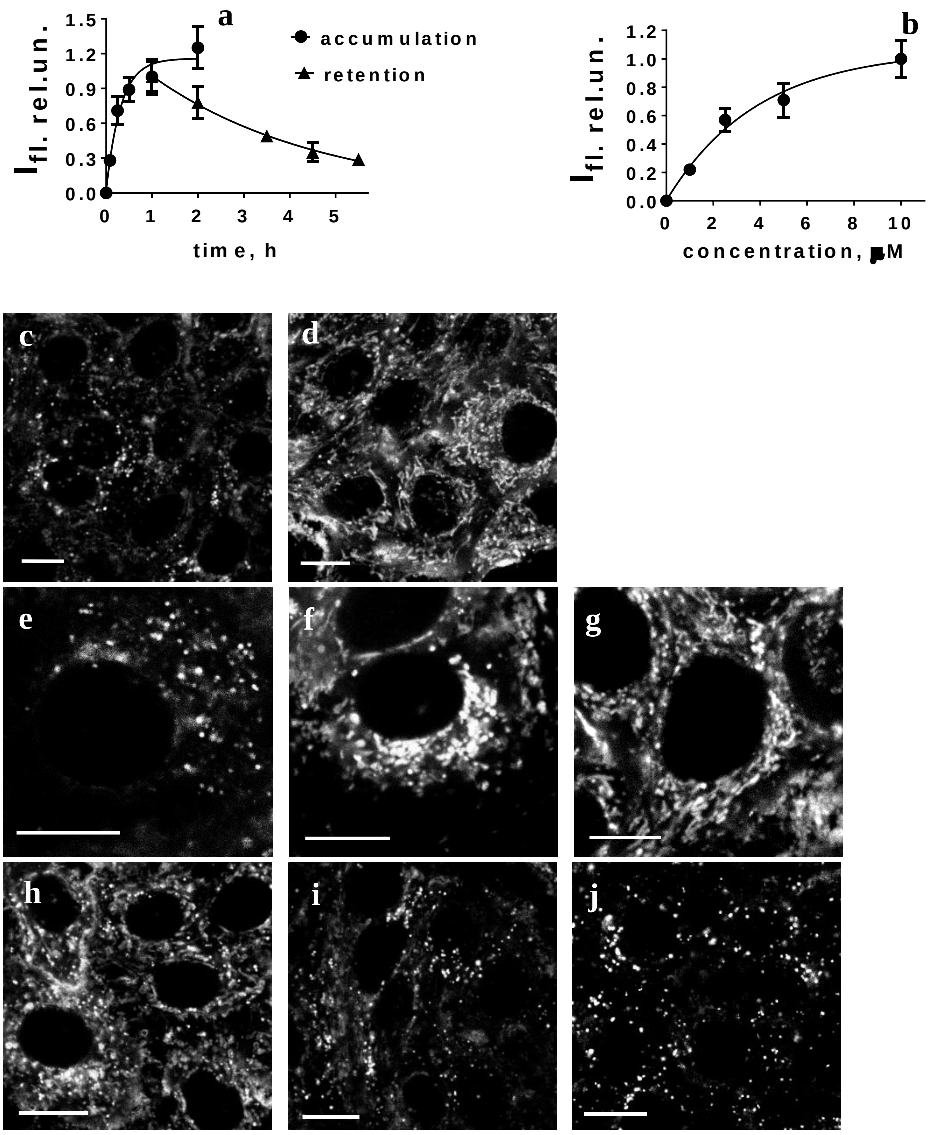

Figure S18. Uptake and retention of **NI-SP** in the A549 cells that were measured using CLSM.

(**a**) Kinetics of accumulation and retention of **NI-SP** in the A549 cells. To measure kinetics of accumulation, cells were incubated with **NI-SP** (10 μM) during different periods of time and imaged with CLSM at the identical parameters of measurements. To characterize retention of **NI-SP** in cells, cells were incubated with **NI-SP** (10 μM) during 1 h, washed twice with Hanks' solution, placed in the fresh medium without **NI-SP** for different (0–4.5 h) periods of time and imaged using CLSM at the identical parameters of measurements.

(**b**) The concentration dependence of **NI-SP** accumulation in A549 cells, which was measured using CLSM after 0.5 h incubation of cells with different concentrations of **NI-SP**.

(**c, d)** Typical CLSM images of **NI-SP** in the A549 cells after 5 (**c**) and 60 (**d**) min incubation of cells with **NI-SP** (10 μM).

(**e-g**) Typical CLSM images of **NI-SP** in the A549 cells after 30 min incubation of cells with **NI-SP** at 1 **(e),** 2.5 **(f)** and 10 μM **(g)**.

(**h-j**) Typical CLSM images of **NI-SP** in the A549 cells after 1 h **(h),** 2.5 h **(i)** and 3.5 h **(j)** efflux of **NI-SP** from cells. Cells were pre-incubated with **NI-SP** (10 μM) for 30 min and placed in the fresh medium without **NI-SP**. Bars represent 10 μm.

6. Interactions of NI-SP with Hg^{2+} in cells: analysis at λ_{ex} = 488 nm

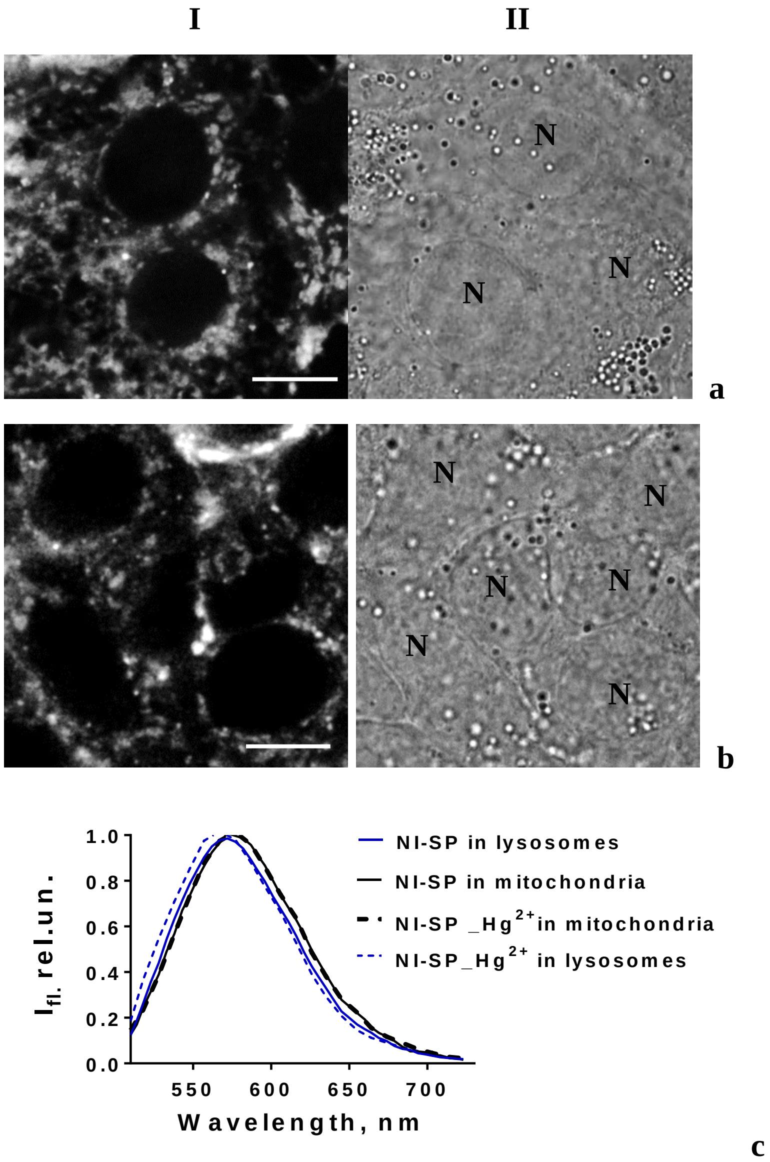

Figure S19. Confocal fluorescent images (**a, b**) and fluorescence spectra (**c**) of **NI-SP** and (**NI-SP**)·Hg^{2+} in A549 cells at the 488 nm excitation wavelength. (**a**) Cells were incubated with **NI-SP** (10 µM) for 20 min. (**b**) Cells were pre-incubated with 20 µM of $Hg(ClO4)_2$ for 20 min, washed twice with Hanks' solution, and incubated with 10 µM of **NI-SP** for 20 min. (**I**) Images of intracellular fluorescence were measured in the 510–730 nm spectral ranges. (**II**) Transmitted-light images of the cells. Bar represents 10 µm. N marks a nucleus. (**c**) Typical normalized intracellular fluorescence spectra of **NI-SP** and (**NI-SP**)·Hg^{2+}.

7. Plot of the ratio *R′*$_{av}$ *versus* increasing concentrations of Hg^{2+} in cells

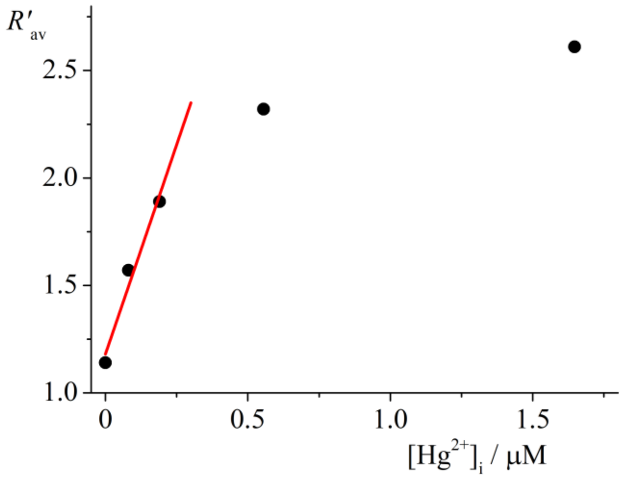

Figure S20. Plot of the ratio R'_{av} for sensor **NI-SP** versus increasing intracellular concentrations of Hg^{2+}. Excitation wavelength is 405 nm.

8. Kinetic data on intracellular accumulation of Hg^{2+}

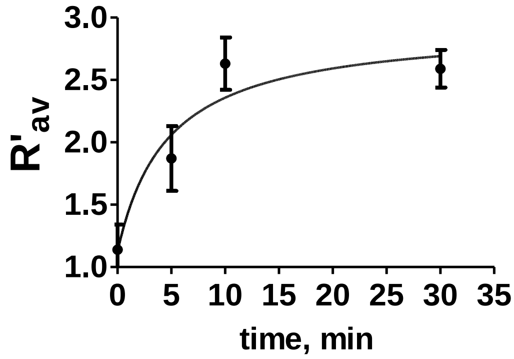

Figure S21. Uptake of Hg^{2+} in the A549 cells measured with **NI-SP**. Cells were pre-incubated with **NI-SP** (10 µM, 20 min), washed and exposed to 10 µM $Hg(ClO_4)_2$ for different periods of time.

9. Interactions of NI-SP with Cu^{2+}, Pb^{2+} and Ag^{+} in living cells

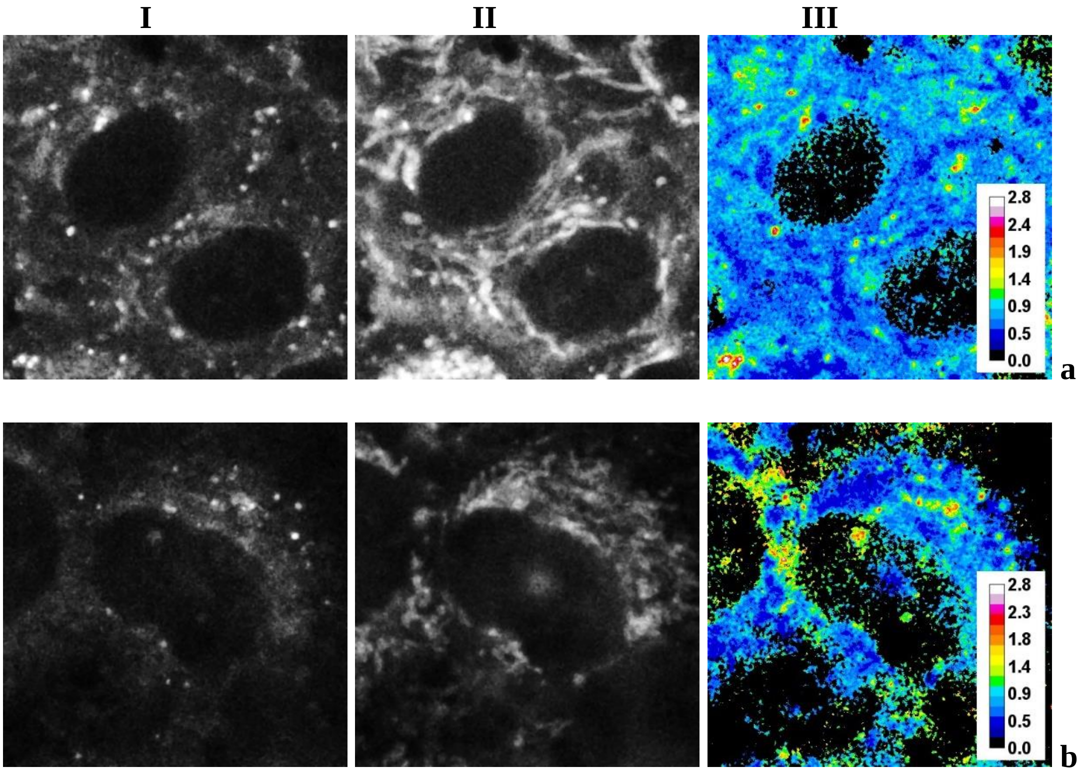

Figure S22. Fluorescence "image maps" of intracellular complexation of **NI-SP** with Cu^{2+} (**a**) and Pb^{2+}(**b**). (**I**)- Images of intracellular fluorescence were measured in the 440–510 spectral range. (**II**)- Images of intracellular fluorescence were measured in the 590–650 nm range. (**III**)- the false-color images showing the ratio R' of the fluorescence intensities $I_{440-510}$ / $I_{590-650}$ at 1 mM of $Cu(ClO_4)_2$ (**a**) and 10 mM of $Pb(ClO_4)_2$ (**b**). Intensity scale corresponds to R' values. The A549 cells were pre-incubated with $Cu(ClO_4)_2$ (1 mM) or $Pb(ClO_4)_2$ (10 mM) for 30 min, washed twice with Hanks' solution, and incubated with **NI-SP (**10 μM) for 20 min.

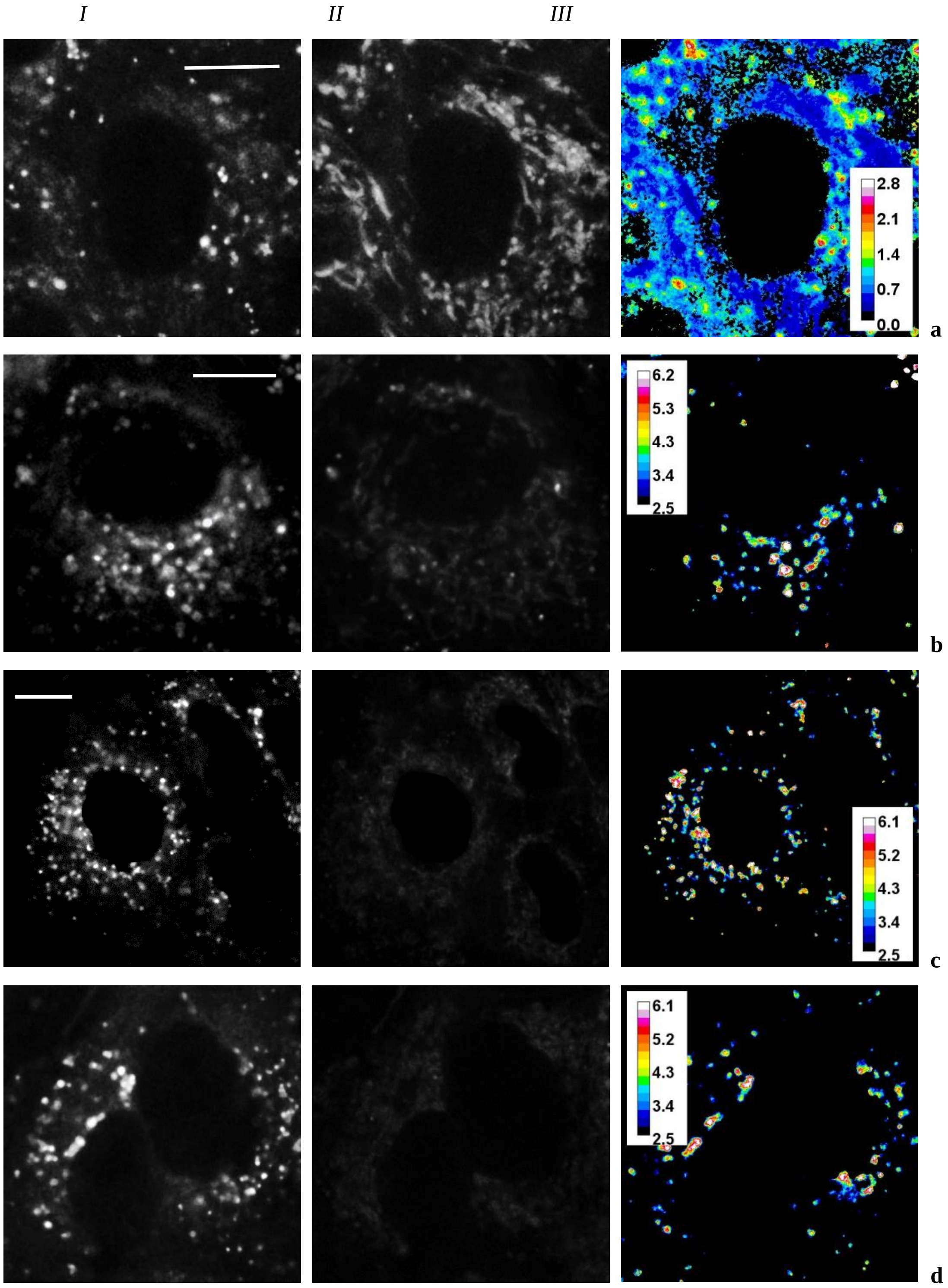

Figure S23. Intracellular distribution of **NI-SP** fluorescence for cells treated with 200 µM $Ag(ClO_4)$ (**a**); with 200 µM $Ag(ClO_4)$ and 20 µM $Hg(ClO_4)_2$ (**b**); with 20 µM $Hg(ClO_4)_2$ (**c**); preincubated with 20 µM $Hg(ClO_4)_2$ and after with 200 µM $Ag(ClO_4)$ (**d**). Fluorescence was excited at 405 nm and measured in the 440 – 510 nm (column *I*) and the 590 – 650 nm (column *II*) spectral ranges. Column *III* shows the ratio R' of the fluorescence intensities $I_{440-510} / I_{590-650}$. Intensity bar is the ratio R' in every point of a cell. Cells A549 were pre-incubated with $Ag(ClO_4)$, or $Hg(ClO_4)_2$ or with $Ag(ClO_4)$ and $Hg(ClO_4)_2$ for 20 min, washed twice with Hanks' solution, and incubated with 10 µM of **NI-SP** for 20 min. Cells pre-incubated with $Hg(ClO_4)_2$ were additionally incubated with $Ag(ClO_4)$ (d).
Bar represents 10 µm. In all the cases the presence of $Ag(ClO_4)$ did not affected the ratio R' in cells. The R'_{av} were equal to 1.03±0.12 in the absence of studied ions; 1.06±0.07 in the presence of 200 µM $Ag(ClO_4)$ (**a**); 2.6±0.3 in the presence of 200 µM $Ag(ClO_4)$ and 20 µM $Hg(ClO_4)_2$ (**b**); 2.6±0.3 in the presence of 20 µM $Hg(ClO_4)_2$ (**c**); 2.6±0.3 in cells preincubated with 20 µM $Hg(ClO_4)_2$ and after with 200 µM $Ag(ClO_4)$ (**d**).

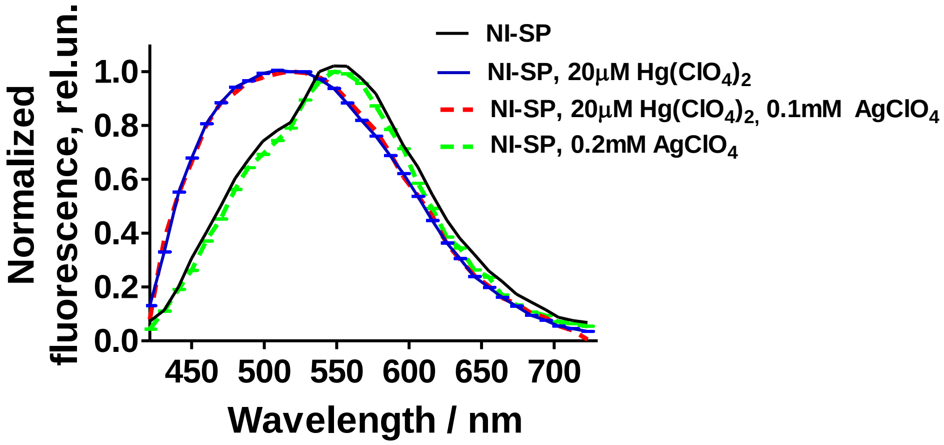

Figure S24. Typical normalized fluorescence spectra of **NI-SP** in lysosomes of cells treated with Ag(ClO_4), Hg$(ClO_4)_2$ or with a mixture of Ag(ClO_4) and Hg$(ClO_4)_2$. Cells were pre-incubated (20 min) with 20 μM Hg$(ClO_4)_2$, or with 0.2 mM Ag(ClO_4), or with a mixture of 20 μM Hg$(ClO_4)_2$ and 0.1 mM Ag(ClO_4), washed twice with Hanks' solution, and incubated with 10 μM of **NI-SP** for 20 min.

10. Cytotoxicity of NI-SP and Hg$(ClO_4)_2$

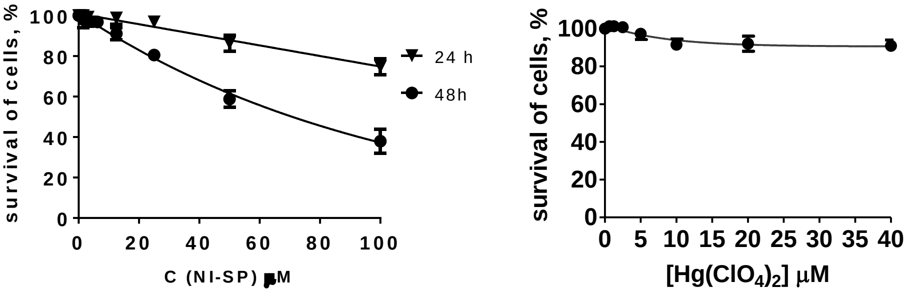

A **B**

Figure S25. Survival of A549 cells at different concentrations of **NI-SP (A)** or Hg$(ClO_4)_2$ (**B**) according to MTT assay. The cells were incubated with **NI-SP** or Hg(ClO4)2 for 24 h or 48 h.

10. NMR and mass-spectra of the synthesized compounds

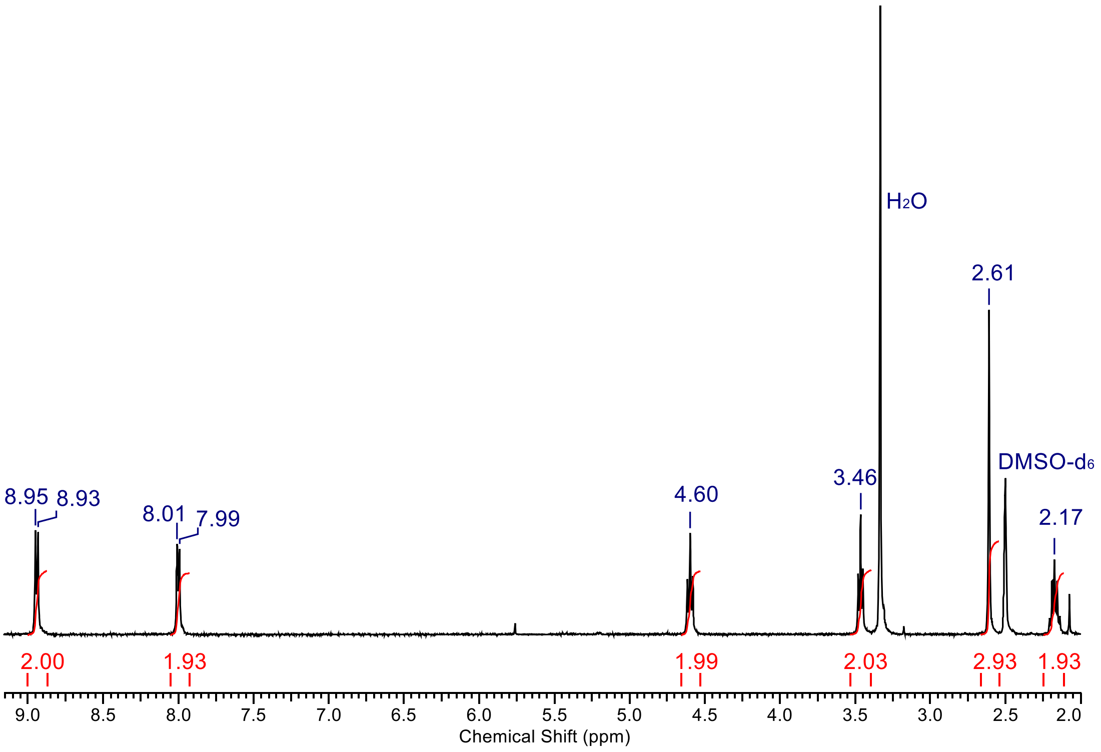

Figure S26. ^{1}H NMR-spectrum of compound **4** in DMSO-d_6.

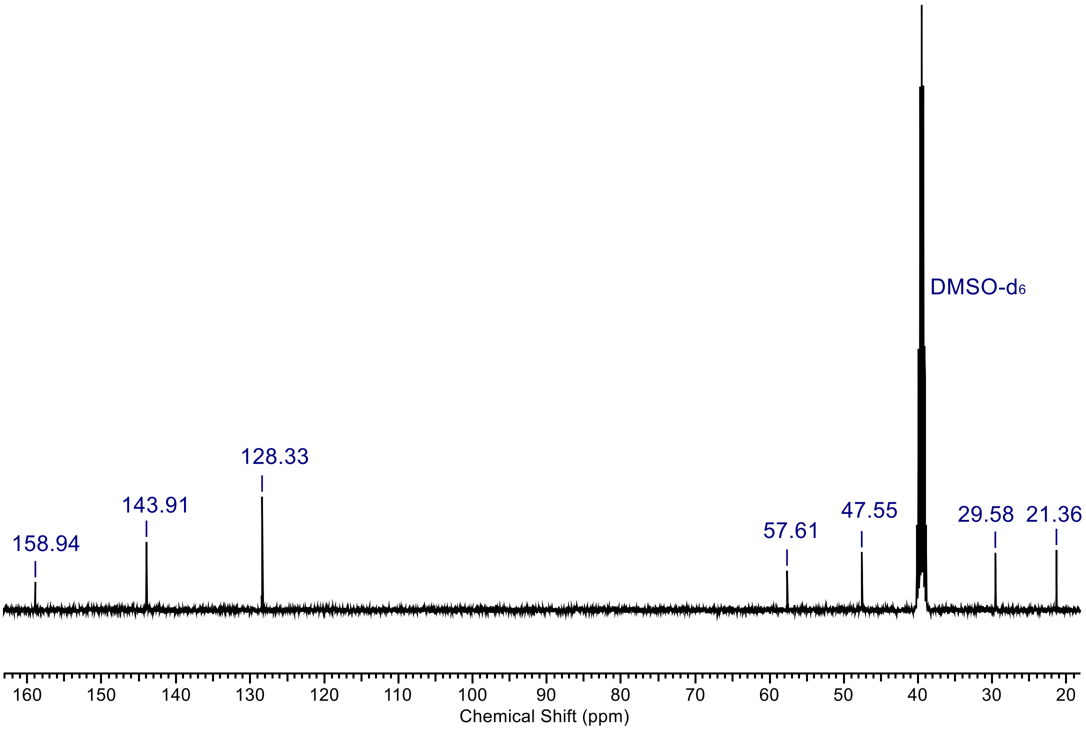

Figure S27. ^{13}C NMR-spectrum of compound **4** in DMSO-d_6.

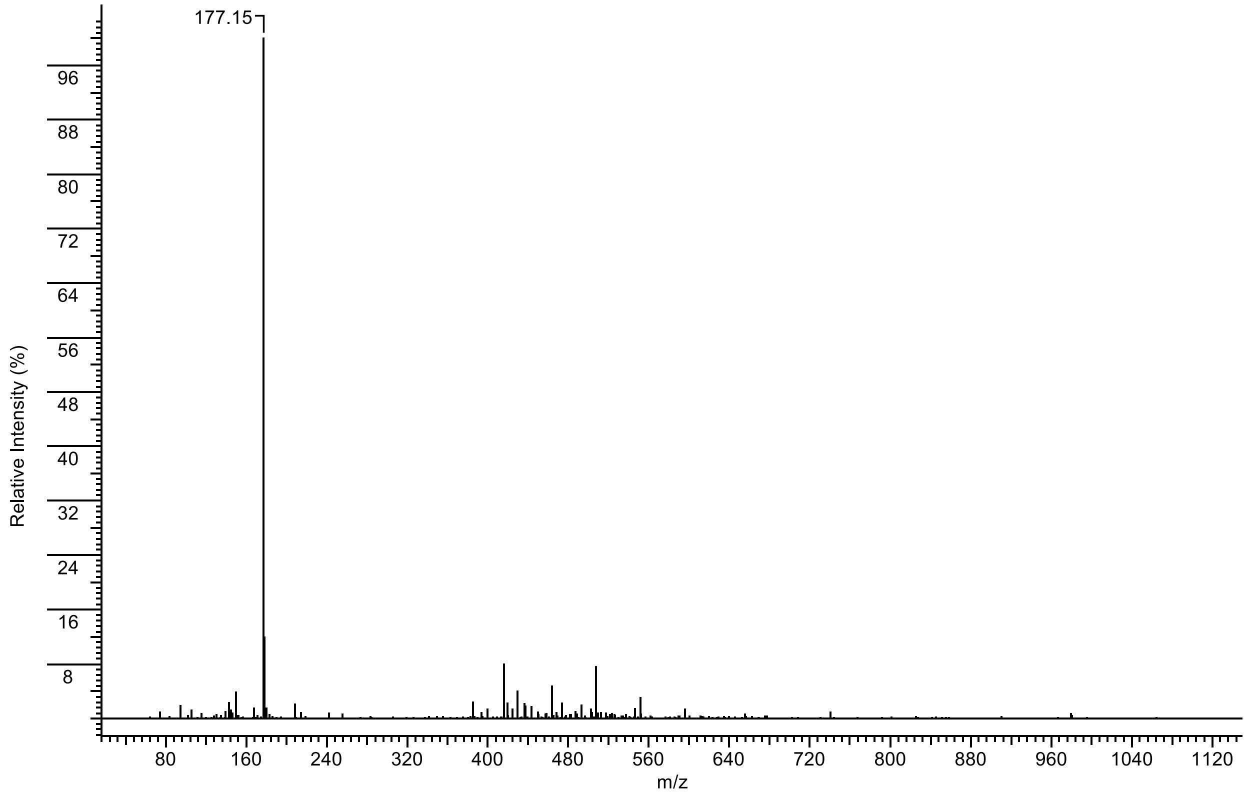

Figure S28. ESI-MS-spectrum of compound **4** in MeOH.

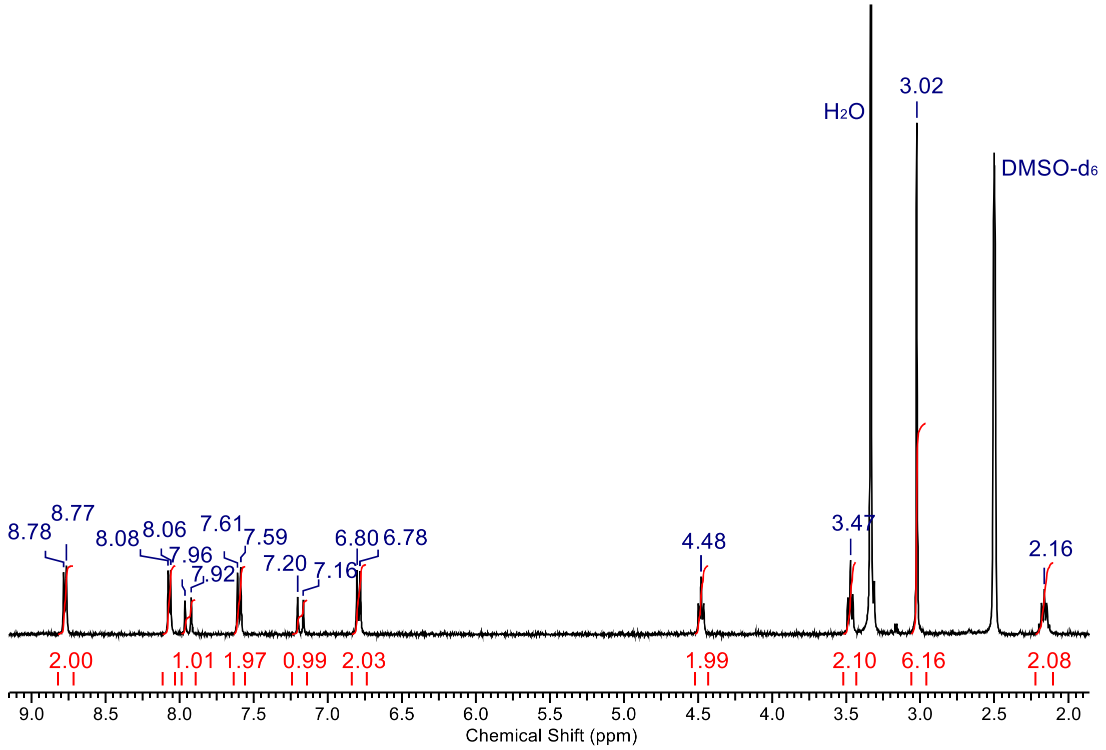

Figure S29. ^{1}H NMR-spectrum of compound **6** in DMSO-d_6.

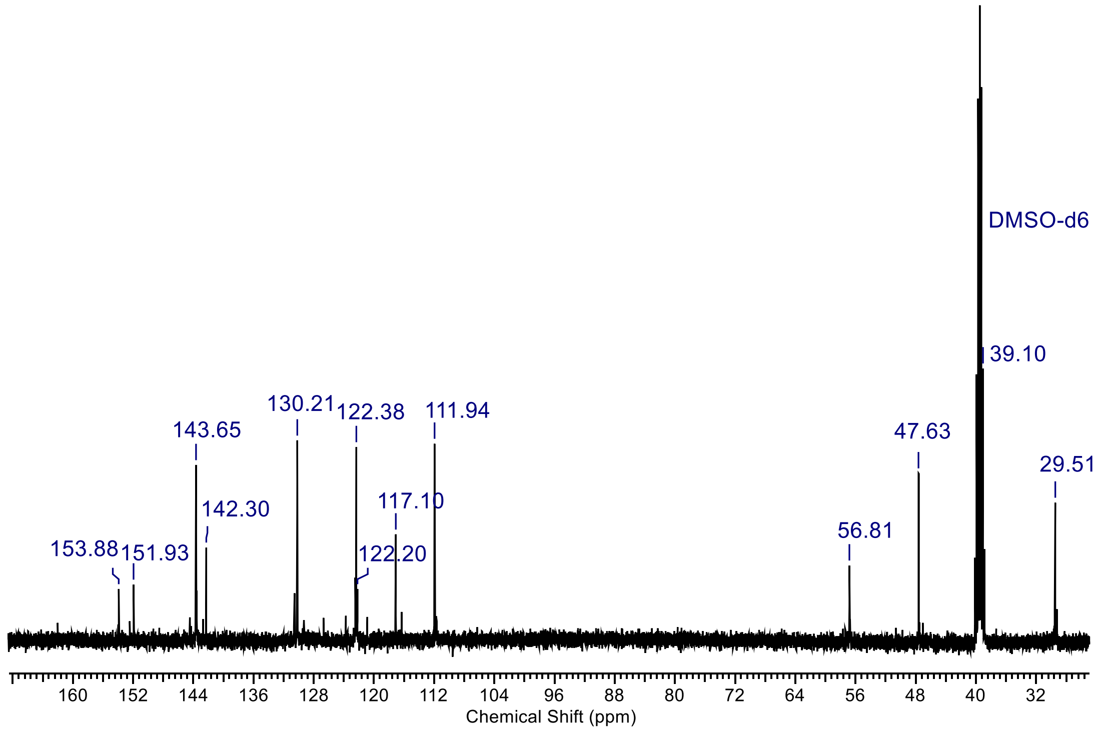

Figure S30. ^{13}C NMR-spectra of compound **6** in DMSO-d_6.

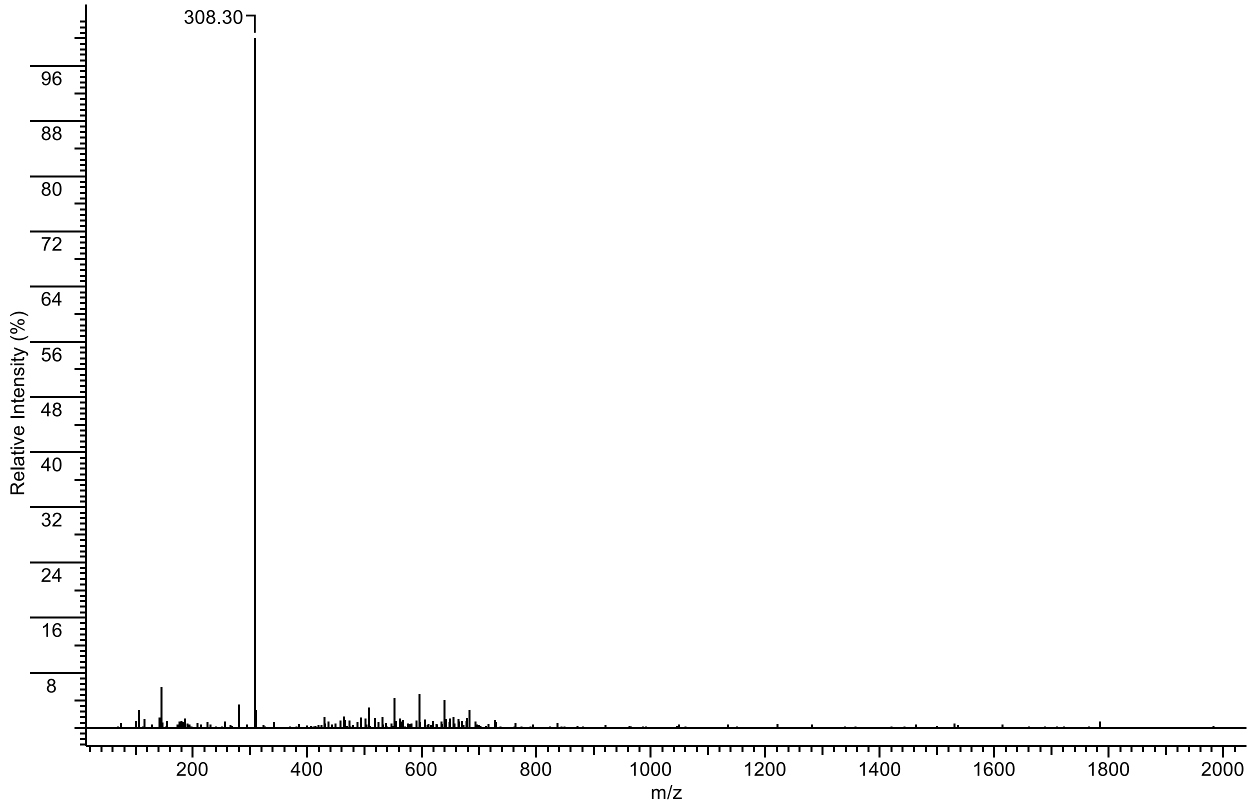

Fig. S31. ESI-MS-spectra of compound **6** in MeOH.

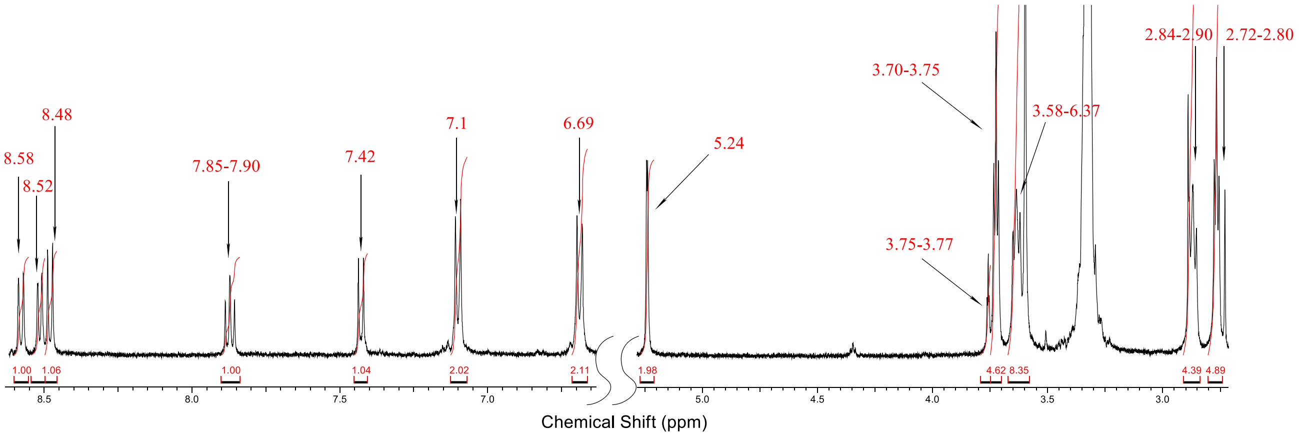

Figure S32. ^{1}H NMR-spectrum of compound **11** in DMSO-d_6.

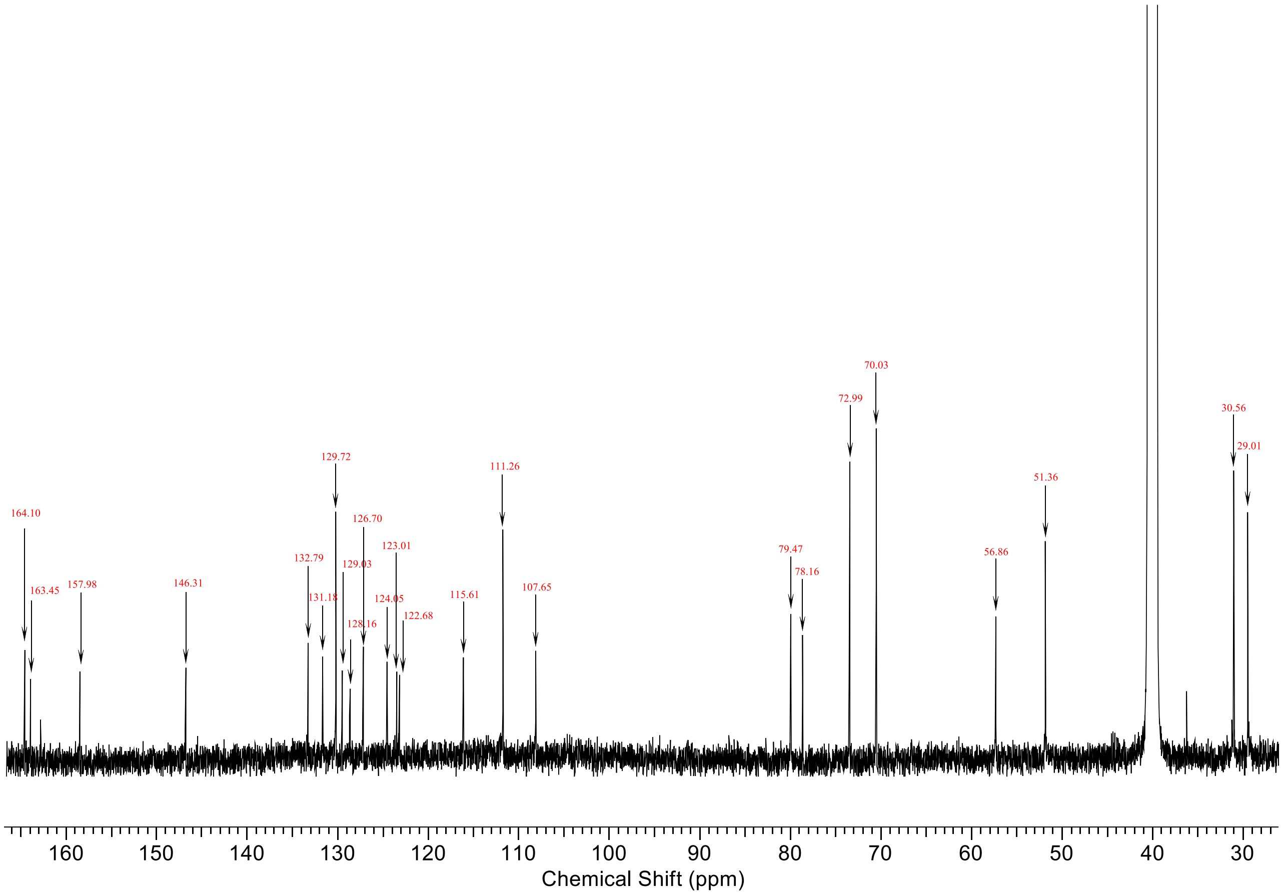

Figure S33. ^{13}C NMR-spectra of compound **11** in DMSO-d_6.

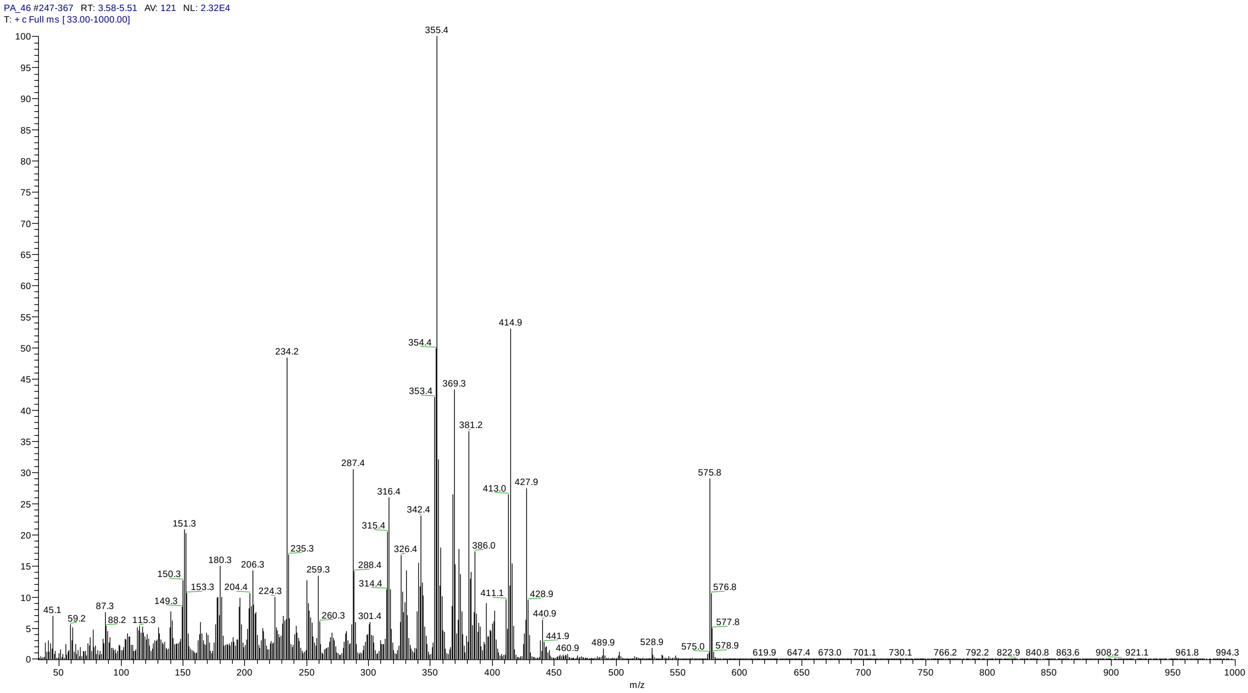

Figure S34. EI-MS-spectrum of compound **11**.

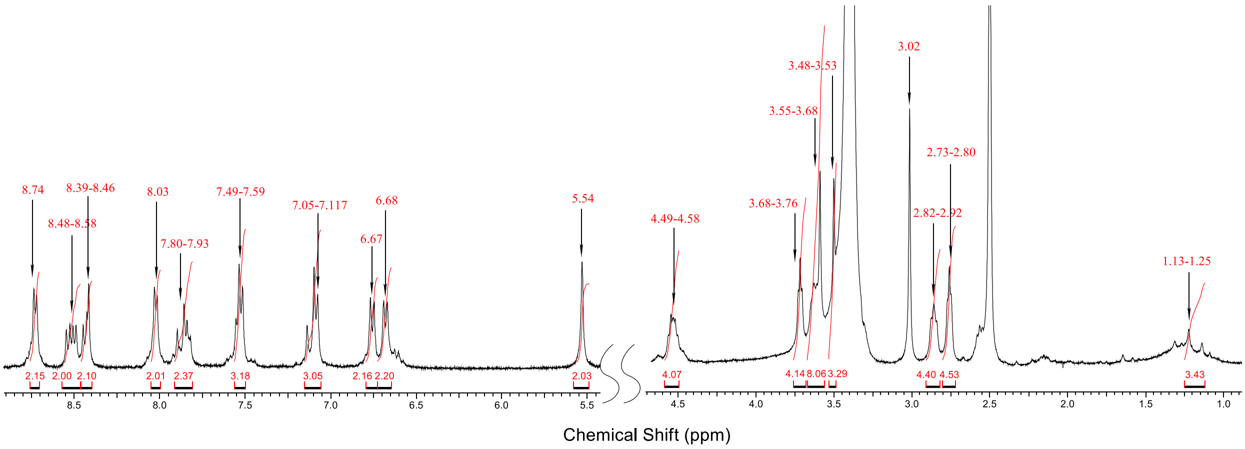

Figure S35. ^{1}H NMR-spectrum of compound **NI-SP** in DMSO-d_6.

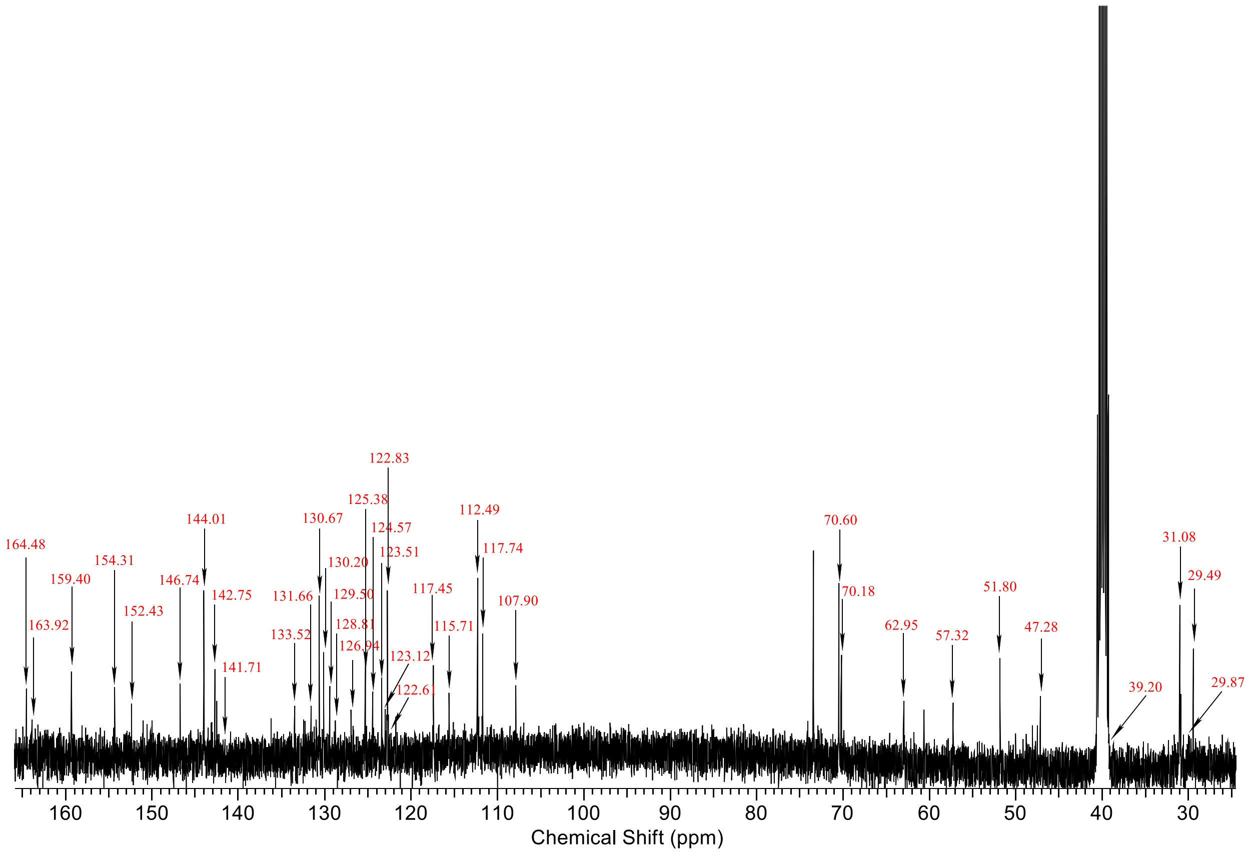

Figure S36. ^{13}C NMR-spectra of compound **NI-SP** in DMSO-d_6.

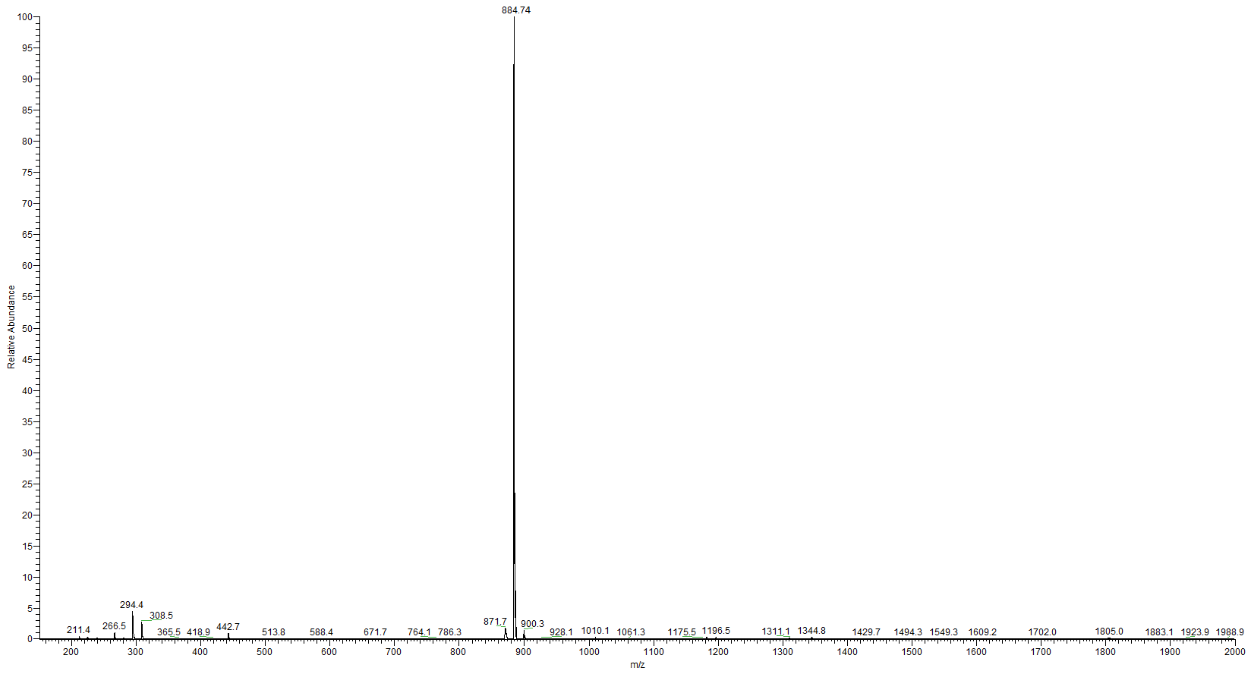

Figure S37. ESI-MS-spectrum of compound **NI-SP** in MeCN.
